# Stemness of the hybrid Epithelial/Mesenchymal State in Breast Cancer and Its Association with Poor Survival

**DOI:** 10.1371/journal.pone.0126522

**Published:** 2015-05-28

**Authors:** Anne Grosse-Wilde, Aymeric Fouquier d’Hérouël, Ellie McIntosh, Gökhan Ertaylan, Alexander Skupin, Rolf E. Kuestner, Antonio del Sol, Kathie-Anne Walters, Sui Huang

**Affiliations:** 1 Institute for Systems Biology, Seattle, WA 98109, United States of America; 2 Luxembourg Centre for Systems Biomedicine, L-4362 Esch-sur-Alzette, Luxembourg; Tel Aviv University, ISRAEL

## Abstract

Breast cancer stem cells (CSCs) are thought to drive recurrence and metastasis. Their identity has been linked to the epithelial to mesenchymal transition (EMT) but remains highly controversial since—depending on the cell-line studied—either epithelial (E) or mesenchymal (M) markers, alone or together have been associated with stemness. Using distinct transcript expression signatures characterizing the three different E, M and hybrid E/M cell-types, our data support a novel model that links a mixed EM signature with stemness in 1) individual cells, 2) luminal and basal cell lines, 3) *in vivo* xenograft mouse models, and 4) in all breast cancer subtypes. In particular, we found that co-expression of E and M signatures was associated with poorest outcome in luminal and basal breast cancer patients as well as with enrichment for stem-like cells in both E and M breast cell-lines. This link between a mixed EM expression signature and stemness was explained by two findings: first, mixed cultures of E and M cells showed increased cooperation in mammosphere formation (indicative of stemness) compared to the more differentiated E and M cell-types. Second, single-cell qPCR analysis revealed that E and M genes could be co-expressed in the same cell. These hybrid E/M cells were generated by both E or M cells and had a combination of several stem-like traits since they displayed increased plasticity, self-renewal, mammosphere formation, and produced ALDH1+ progenies, while more differentiated M cells showed less plasticity and E cells showed less self-renewal. Thus, the hybrid E/M state reflecting stemness and its promotion by E-M cooperation offers a dual biological rationale for the robust association of the mixed EM signature with poor prognosis, independent of cellular origin. Together, our model explains previous paradoxical findings that breast CSCs appear to be M in luminal cell-lines but E in basal breast cancer cell-lines. Our results suggest that targeting E/M heterogeneity by eliminating hybrid E/M cells and cooperation between E and M cell-types could improve breast cancer patient survival independent of breast cancer-subtype.

## Introduction

Poor cancer patient survival has been linked to enrichment for cancer stem cells (CSCs) [1] that are capable of undergoing the “metastatic cascade”, including invasion, migration, survival in suspension, colonization and establishment of secondary tumors [2]. Thus, identification of single CSCs by yet unknown markers promises to enable prediction of patient outcome, as well as to facilitate targeting of therapy to these cells to improve patient survival. CSCs (or alternatively ‘tumor-initiating cells‘), like normal stem cells, are thought to be capable of self-renewal and plasticity, leading to heterogeneous progeny [3]. Due to their plasticity, normal mammary epithelial stem cells give rise to both luminal and basal (myoepithelial) lineages [4]. Evidence is accumulating that all breast cancer cells are derived from a common luminal stem-like cell population that gives rise to both luminal and basal tumors [5–8]. A tight link between luminal estrogen receptor (ER)+ and basal ER- breast tumors is also suggested by the observation that antihormonal treatment of ER+ breast cancer patients with tamoxifen treatment increases risk of contralateral development of ER- tumors [9]. While many prognostic signatures have been identified, all of them predict poor patient outcome either in luminal ER+ or in basal ER- tumors [10], thus requiring the context (microenvironment) of the particular tumor type to be predictive, they are agnostic of the existence of a common CSC population for luminal and basal breast cancer patients.

‘Stemness’ of tumor cells is measured *in vitro* by their ability to form mammospheres [11] and *in vivo* by their tumor-initiation capability in immune-compromised mice. Mammosphere formation, tumor initiation, as well as the early steps of the metastatic process that require survival of the disseminating cells as circulating tumor cells (CTCs) can be induced by an epithelial-to-mesenchymal transition (EMT), which affords epithelial (E) tumor cells a mesenchymal (M) phenotype [12–15]. Therefore, M cells are often considered CSCs, and E cells are considered ‘non-CSCs’ [16]. However, this simple dualism remains controversial for several reasons: first, tumor initiation at metastatic sites requires epithelial gene expression, implying a mesenchymal-to-epithelial transition (MET) during later steps of the metastatic cascade facilitates colonization [17]. Second, recent findings show that expression in primary tumors of epithelial markers [18–20] but not mesenchymal markers [19,21,22] predict metastasis and poor outcome in breast cancer patients. Third, in many cell-lines, CSC-related properties, such as migration or formation of mammospheres and increased tumorigenicity are often not associated with expression of mesenchymal genes but rather with enrichment for epithelial gene expression in breast cancer [20,23,24] as well as in other carcinoma [23,25–27]. Therefore, CSC properties are not necessarily associated with the M phenotype but sometimes also with cells that have the opposite, more E-like phenotype and express more E-specific genes. In brief, studies in breast cancer patients, in cancer cell-lines *in vitro* and in xenograft animal models suggest that the CSC characteristic is not a property that is intrinsically associated with the mesenchymal state but must also bear some relationship with the epithelial state.

We noticed that in many cancerous breast cell-lines with the luminal (epithelial) phenotypes, such as HMLE, HMLER, and MCF7 cells [12–14]_,_ the CSC-enriched population was consistently associated with high expression of M genes, whereas in cell-lines with basal (mesenchymal) morphology, such as BT-549, MDA-MB231, or 4T07_,_ the CSC-enriched population expressed more E genes [20,23,24]. This suggests that the context for E molecular marker signatures to indicate stemness may be the M tumor cell-lines, whereas conversely, the M marker signatures indicate stemness when expressed in E tumor cell-lines. Given the plasticity of both normal stem cells and cancer stem-like cells that can give rise to both luminal and basal lineages it is possible that a common signature can identify the CSC-enriched populations in breast cancer cells and in patients—independent of the cell line or tumor-context being epithelial, mesenchymal, luminal, or basal. This signature could appear epithelial relative to the mesenchymal bulk tumor cells, but mesenchymal in the context of mostly epithelial tumor cells.

Therefore we hypothesized that stemness is associated with cells exhibiting a hybrid E/M state, cells simultaneously expressing both E and M markers. This hybrid state is possibly the result of an ‘incomplete EMT’ for E cells and an ‘incomplete MET’ for M cells as suggested previously [5,28,29]. In stem cell biology the concept of promiscuous gene expression patterns in bi-potential progenitor cells has long been proposed on theoretical grounds and demonstrated in *in vitro* models that display a promiscuous phenotype between the prospective lineages fates [30,31]. Indeed, in xenograft animal studies, it has been observed that small populations of CSCs for example isolated from breast [32–34] and ovarian cancer [35] co-expressed different E (EPCAM, CD24, or ALDH) and M (CD44) cell surface markers.

However, several questions remain open: (1) the “extreme” (pure) E and M states as well as the intermediate E/M state have not been explicitly described yet by the expression of definite sets of genes which have been very inconsistent between different tumor cell lines. (2) With regard to the metastatic potential and poor survival in ER+ and ER- breast cancer patients there is still no evidence for the functional relevance of hybrid E/M state. (3) In whole-tissue gene expression profiles that are routinely measured, the ‘co-expression’ of E and M markers may be the consequence of a mixture of relatively differentiated E and M cells as opposed to the presence of individual stem-like cells in the postulated hybrid E/M state.

## Results

### Identification of luminal epithelial and basal mesenchymal genes in HMLER cells

**Fig 1:**
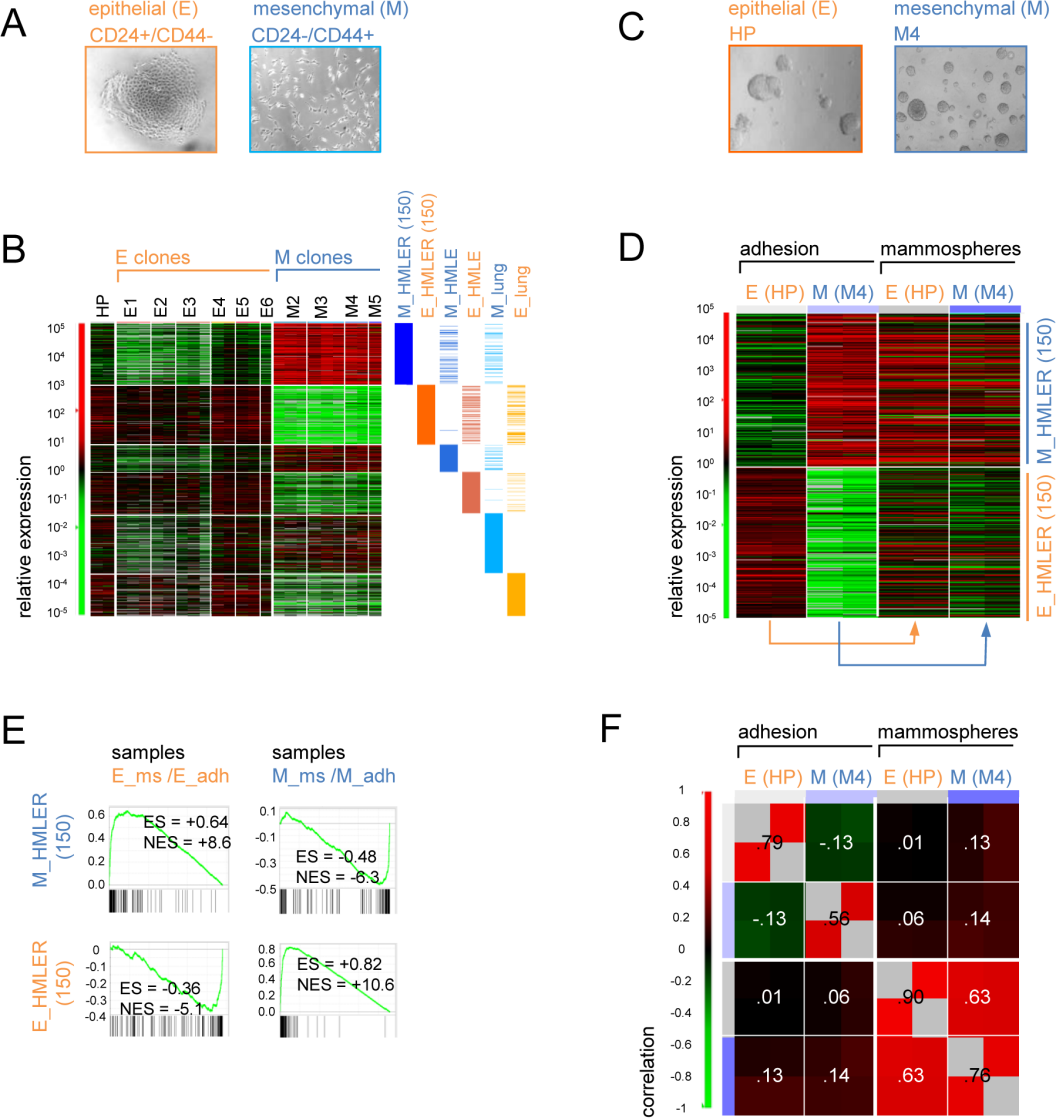
In mammospheres E and M cell lines converge to an intermediate E/M gene expression state. (A) Phase-contrast (20x) images showing a typical HMLER CD24+/CD44-(E) clone and a typical HMLER CD24-/CD44+(M) clone. Orange color refers to epithelial cells, and blue color to mesenchymal cells throughout manuscript. (B) Heat map of the E and M-specific gene expression defined as the 150 most extreme differentially expressed genes between morphological E clones and M clones (E_HMLER (150), and M_HMLER (150), S1 Table) in different HMLER cell-lines. RNA for gene expression arrays shown was isolated from different cell line passages. Gene expression data were relative-normalized towards the intermediate expression between E and M clones. Overlap with other E and M gene sets from different cell-types (HMLE cells and lung cancer) is shown on the right. (C) Phase-contrast (20x) images showing mammospheres grown in suspension of epithelial HP cells and the mesenchymal M4 clone. (D) Heat map of E and M-specific genes expressed in HP(E) and M4(M) cells when grown under adhesion and suspension mammospheres conditions. Normalization of gene expression was as in B, biological duplicate arrays are shown. (E) GSEA of gene expression dynamics between cells grown in adhesion *versus* suspension from (D), assessed for the E- and M-specific genes. (Normalized) Enrichment Scores (NES and ES) were based on duplicate arrays, had a false discovery rate q-value of < 0.0001 and a family wise p-value < 0.01 (S3 Table).(F) Pearson correlation coefficient between whole transcriptomes from (D) for global profiles. Numbers in heatmap represent the correlation average.

To discriminate the hybrid E/M state from the more extreme E and M cell states, we first determined the genes most specifically expressed in morphologically E and M cell-types (Fig 1A). To this end we used the well characterized tumorigenic HMLER cells because they contain both epithelial CD24+/CD44- (also previously designated CD24+/CD44lo) and mesenchymal CD24-/CD44+ (CD24-/CD44hi) cell-types that can interconvert into each other [13,36], are genetically mostly identical and thus comparable with each other. HMLER cells are derived from normal human mammary epithelial (HMEC) cells by consecutive immortalization and transformation with *hTERT* and the *SV40LT* and *RAS* oncogenes [37]. By single cell-sorting we generated morphologically stable (> 20 passages) CD24+/CD44-(E) clones and CD24-/CD44+(M) clones (S1 Fig); E clones had the typical epithelial cobble stone-like morphology with tight cell-cell contacts, while M clones grew rather as dispersed cells and had fibroblast-like morphology (Fig 1A). To derive the most differentially expressed genes between these opposite E and M phenotypes, we compared transcriptomes of six E and four M clones with each other. E and M-specific genes were ranked by their differential expression in the E clones *versus* M clones (designated E_HMLER and M_HMLER gene sets, S1 and S2 Tables, Fig 1B).

Previously, independent E and M gene sets have been reported by differential overexpression of genes in the parental HMLE cells that promote the E *versus* M type cells. Cells were rendered M by overexpression of either *TWIST*, *SNAIL*, siCDH1, *GSC* or TGFβ treatment, and highly expressed genes (M genes) were designated as ‘EMT core signature’ [22]. HMLE cells are less tumorigenic and not transformed with the *RAS* oncogene as HMLER cells. HMLE E and M gene sets substantially overlapped with the E_HMLER and M_HMLER signatures but also contained unique genes (Fig 1B, S2 Table, S1 Fig). Similarly, lung-specific E and M signatures [38] (S2 Table) overlapped significantly with HMLER- and HMLE-derived E and M signatures (Fig 1B), indicating that the E and M states defined by these genes are not specific for *RAS* expression nor only breast-specific but associated with the respective opposite cellular morphologies. As expected, among the most extremely expressed E_HMLER genes were well-known E genes such as *CDH1* (E-Cadherin), *EPCAM* (ESA), and *CD24*. The set of M_HMLER genes contained connective tissue-specific genes such as *VIM* (Vimentin), *DCN* (Decorin) and genes encoding several collagens and were characteristic for the mesenchymal-like ‘basal B’ cell-lines (S1 and S2 Tables). With respect to cell-lines derived from primary breast tumors, according to the online GOBO tool [39] based on classification by Neve [40], E_HMLER and M_HMLER genes correlated either with the luminal (epithelial) or basal B (mesenchymal) patient-derived cancer cell-lines, respectively (S1B Fig). This suggested that – relative to each other – the HMLER-derived E and M gene sets indeed represent the typical cellular program found in the more luminal-like and basal-like breast cancer cell-lines, respectively.

### Mammospheres from E and M cell lines converge to an intermediate E/M gene expression state

Mammospheres are not only derived from stem-like cells [11], but also enriched for cells with stem-cell properties, such as tumorigenicity [41,42]. Thus, transcriptomes of mammospheres may also show enrichment for genes characteristic of CSCs. Therefore, we analyzed the transcriptomes of E and M HMLER cell populations grown in adhesion and as mammospheres in suspension (Fig 1C). As representative for HMLER E populations we used the epithelial parental HMLER cells (here: HP cells, mostly CD24+/CD44- cells, <30% CD24-/CD44+ cells), since in adhesion HP cells showed E-clone like transcriptomes with respect to E and M genes (Fig 1B). (E clone E5 mammospheres did not yield sufficient RNA for transcriptome analyses). Conversely, as representative HMLER M population we used the M4 clone. We observed that the majority of genes whose expression increased following the transition from adhesion to mammosphere suspension cultures differed in the E and M cells, and thus depended on the cell-type. However, about 15% of transcripts changed in a context-independent manner, i.e., were altered in both cell-lines (S2A Fig). Consistent with the reported enrichment for stem-like cells, mammospheres from both, the HP and M4 cells displayed increased expression levels of transcripts for the pluripotency factor OCT4 (*POU5F1*) relative to adherent cells (S2B Fig). Also, both the HP and M4-derived mammospheres showed an increase of *APOE* and loss of *NRG1* expression as previously observed in mammospheres derived from normal breast HMEC cells [11]. This suggested that, as in normal breast cells, both E and M HMLER cell populations adopt a stem-like phenotype when grown as mammospheres.

In striking contrast to these cell-type-independent markers of stemness, the E- and M-specific genes changed in exactly opposing directions depending on the cell-type context, E (HP) vs M (M4) cells (Fig 1D and S2A Fig). Specifically, epithelial HP cells exhibited a significant increase of M-specific transcripts and some loss of E-specific transcripts as confirmed by gene set enrichment analysis (GSEA) (Fig 1D and 1E) for signatures from both HMLER and HMLE cells (S3 Table). This observation is consistent with the conventional notion of a link between stemness of the cells in mammospheres and the mesenchymal phenotype [13,14]. Of note, the transcriptomes of E (HP) cell-derived mammospheres did not completely convert to the transcriptomes of adherent M (M4) cells but rather displayed an intermediate EM expression pattern, suggesting an incomplete E to M conversion (Fig 1D). Intriguingly, the transcriptomes of the M cell-derived mammospheres changed in the *opposite* direction and also converged to an intermediate EM profile. Specifically, in M cell-derived mammospheres, expression of E-specific transcripts was increased and M-specific transcripts decreased (Fig 1D and 1E). These observations would be consistent with the notion of incomplete M to E conversion (MET) in M4 cells, and of incomplete EMT in HP cells.

The formation of an intermediate EM gene expression pattern upon transfer from adhesion to suspension (mammospheres) culture involved convergent gene expression shifts at two levels: (1) at the level of E and M-specific genes, manifest in the change from anti-correlated in adherent E versus M cells (Pearson correlation coefficient, r = -0.44) to highly correlated in mammospheres (r = +0.82) (S2C Fig); (2) at the level of the whole transcriptome (‘all genes’) from anti-correlated in adherent E versus M cells (*r* = -0.13) to correlated (*r* = +0.63) in mammospheres (Fig 1F).

Together, our data confirm that—like in other luminal epithelial cancer cell lines – HP cells with highest stemness induced by mammosphere formation displayed a mesenchymal gene expression program. By contrast, for M4 cells—like in basal mesenchymal cancer cell lines—the stem-like cells enriched in mammospheres expressed more epithelial genes. Due to this context-dependent increase of expression of either M or E-specific genes in mammospheres and the incomplete transition to the opposite state the stem cell-enriched mammospheres from both HP (E) and M4 (M) cells exhibit co-expression of both E and M genes, confirming our hypothesis of stemness of the intermediate state in HMLER cell lines.

### Co-expression of E and M genes predicts poor outcomes in all breast cancer subtypes

If the mixed E and M signatures seen in the mammospheres are associated with stemness they may be able to predict poor survival in breast cancer patients. We therefore analyzed a publicly available database of primary breast tumors in up to 3,455 patients using the Kaplan-Meier plotter tool [43]. We sub-classified the primary breast tumors using the St. Gallen clinico-pathological criteria into four breast cancer subtypes: the epithelial-like ER+ slow proliferating luminal A and fast proliferating luminal B subtypes, the mesenchymal-like ER- basal and HER2 positive subtype (see methods).

**Fig 2:**
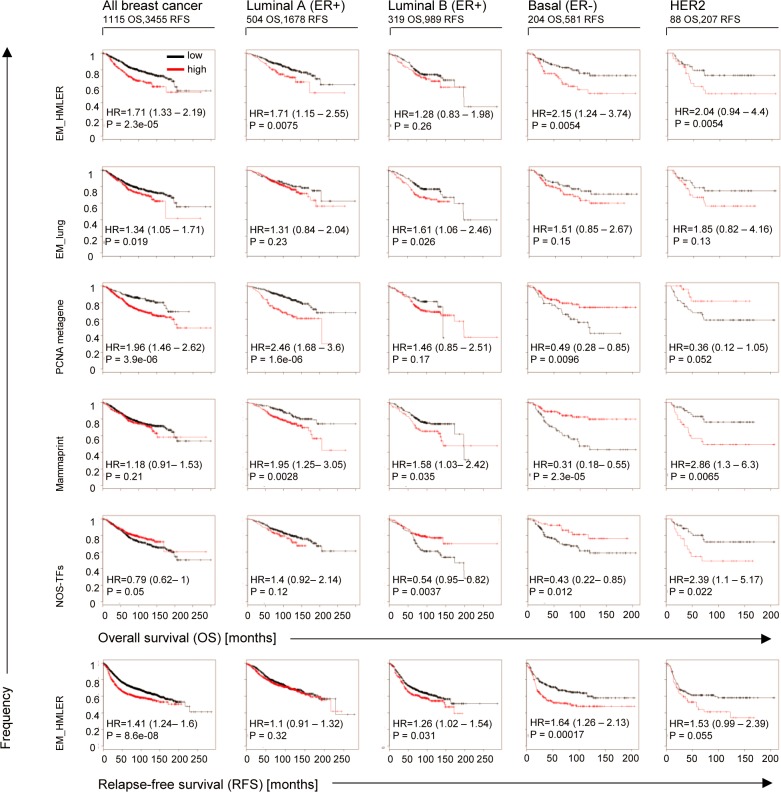
Co-expression of E and M genes predicts poor breast cancer patient survival. Kaplan-Meier plots showing time of overall survival (OS) and relapse-free survival (RFS) of breast cancer patients with different intrinsic breast cancer subtypes using the indicated gene expression signatures consisting of 60 genes (S2 Table) and the Kaplan-Meier-Plotter online tool (database 2014). Numbers of patients within tumor subtype for OS or RFS are indicated on top. Hazard ratio (HR) and respective logrank p-values (P) that discriminate high (red line) and low (black line) expressing patient groups are shown.

To identify the intermediate E/M state, we generated an ‘EM’ signature consisting of 60 genes composed of the respective 30 most extremely expressed E and 30 M genes in HMLER cells (S2 Table, hereafter termed ‘EM_HMLER’), as opposed to the E state described by E genes and the M state described by only M genes alone. Intriguingly, we found that the EM_HMLER signature predicted poor overall survival in the ‘all’ breast cancer category as well as in all four breast cancer subtypes analyzed, which all had a high hazard ratio (HR>1), all with significant logrank p-values except for the luminal B patients subgroup (Fig 2). Accordingly, in all four subtypes as well as in the ‘all’ breast cancer category expression of EM_HMLER was also associated with poor relapse-free survival that was significant for all subtypes except for the rarely metastasizing luminal A and the HER2 subgroup. Also, the EM_lung signature (S2 Table) was associated with poor overall survival in all breast cancer subtypes, but only had a significant p-value for ‘all breast cancer’ and luminal B subtypes. Together, these findings suggested that genes associated with the opposite *morphologies* of E and M cell-types and not only breast-specific E and M genes are associated with poor outcomes in all breast cancer sub-types.

To compare the capability of the EM signatures with other signatures thought to be associated with poor patient outcome, we tested the proliferation-associated PCNA metagene signature [44] and the mammaprint 70 signature [45] (S2 Table). As expected, in the largest group of breast cancer patients with ER+ luminal A breast cancer the PCNA metagene and mammaprint signatures significantly predicted poor survival (Fig 2). However, unlike EM signatures, neither of these signatures was capable to predict poor survival in *all* four breast cancer subtypes. More strikingly, the PCNA metagene signature significantly predicted *good* overall survival in both ER- patient groups (HR<1 for HER2+ and basal breast cancer, p = 0.05 and p = 0.01, respectively); and the mammaprint signature also significantly predicted good survival in basal tumors (p = 10^–5^). Similarly, another signature thought to be associated with stemness, the pluripotency factor-associated signature (NANOG-, OCT4-, SOX2-activated transcription factors, NOS-TFs)[46] also failed to predict poor survival in breast cancer, as it was significantly associated with good survival in ‘all’ breast cancer, luminal B and basal breast cancer patients (p = 0.05, p = 0.004 and p = 0.01, respectively). These data are consistent with previous studies for the NOS-TFs signature showing that it did not correlate with poor outcome in breast cancer [46], and with previous observations that mammaprint and proliferation signatures have only been shown to be prognostic in ER+ and HER2- breast cancer subtypes [10].

Since both the lung and the HMLER-derived E and M gene sets overlapped substantially with the HMLER E and M gene sets, this suggested redundancy in capability of predicting poor survival of EM signatures in breast cancer subtypes. If so, then a smaller subset of the 60 EM_HMLER genes and possibly also overlapping but not identical HMLE-derived EM signatures [22] should also be capable to predict poor outcome. For these analyses we focused only on the more aggressive ER+ subtype luminal B that most frequently metastasizes and the ER- basal breast cancer patients, where mammaprint and PCNA metagene failed to predict poor outcome. From our HMLER-derived E and M signatures and the previously published [22] five different HMLE E and M signatures, we used the 12 most specific E genes and M genes each, to compose a set of EM signatures of 24 genes, named EM_HMLER(24), EM_GSC(24), EM_TWIST(24), EM_ SNAI1(24), EM_siCDH1(24), EM_TGF (24) (S2 Table). The results for Kaplan-Meier analyses are displayed as HR versus logrank p-value plots for overall patient survival (S3A Fig): in basal tumors all six different EM signatures predicted poor survival (HR>0), with four of them associated with a significant p-value. Equally, also in luminal B patients all EM signatures predicted poor survival, five of them significantly. Analysis of relapse-free survival data (S3B Fig) confirmed the same tendency of all EM signatures to predict poor survival in basal and luminal B tumors, and thus all of the only 24 genes comprising EM signatures exceeded the prognostic value of PCNA metagene and mammaprint signatures (Fig 2) to predict poor outcome in both ER+ and ER- breast cancer patients.

Random gene sets often also predict poor outcome, independent of their biological meaning [44]. Therefore we tested if our results for the EM signatures could have been due to chance. We found that, as expected, random gene sets showed similar distributions of hazard ratios <1 and >1 for overall survival in basal and luminal B patients (S3D Fig). By contrast, relative to sets of genes randomly chosen from the whole genome and permutation analysis, all six EM signatures still predicted poor outcome in both patient subtypes with three of them significantly (S3C Fig). Finally, bootstrapping (10,000 different gene sets of 10 out of the 24 most EM-specific HMLER genes) confirmed that the majority of EM genes were associated with poor survival in luminal B and basal patients (S3E Fig).

Together, these data showed that expression of a set of signatures composed of genes associated with the epithelial and the mesenchymal phenotypes (derived from HMLE, HMLER and lung cancer) reliably predicted poor outcome independent of the breast cancer subtype being ER+ or ER-, while NOS-TFs, mammaprint and the PCNA metagene signatures were not predictive or were restricted to ER+ tumors. This suggested that the CSC population responsible for relapse and poor survival of patients of all breast cancer-subtypes is characterized by the intermediate E/M state which is therefore independent of the tumor-type *context*.

### Segregation of plasticity with epithelial cell-types and self-renewal with mesenchymal cell-types during mammosphere formation

The above findings strongly associated a mixed E and M gene expression signature with stemness and aggressiveness in luminal and basal whole-cell populations (Fig 1) and in all breast cancer subtypes (Fig 2). To understand stem cell-relevant properties conferred by co-expression of E and M genes, we dissected which stem-like properties are associated with cells normally mostly expressing E genes *versus* cells expressing M genes in the mammosphere assay. We wished to compare the capacity of ‘self-renewal’ and ‘generation of heterogeneous progeny’ (plasticity) in genetically identical E and M HMLER cell-lines by single cell analyses using flow cytometry and single cell qPCR analysis.

**Fig 3:**
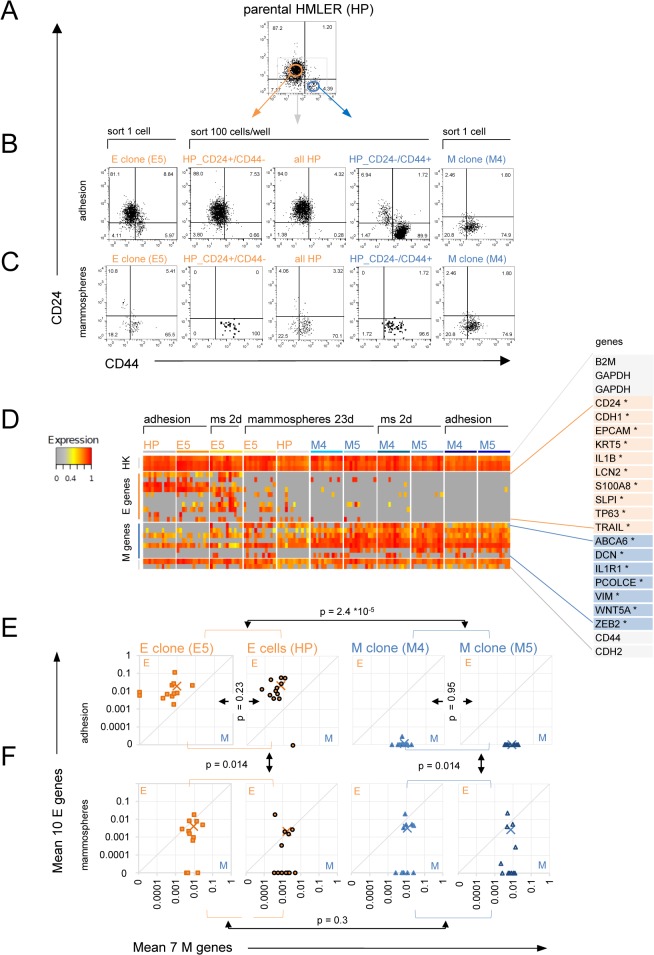
Single cell analysis of EMT and MET state transitions during mammosphere formation. (A) Flow cytometry profiles of the expression of CD24 and CD44 cell surface antigens in adherent grown parental HMLER (HP) cells. Cells were sorted from the indicated gates and (B) cultured in adhesion until confluence or (C) as mammospheres for two weeks in suspension. Cells were individualized, stained for CD24 and CD44 and again analyzed by flow cytometry. Data shown are from biological duplicates and are representative of at least three independent experiments. (D) Heat map of single cell qPCR analysis (BioMark Fluidigm) from sorted cells of E (HP, E5) and M (M4, M5) populations under adherent and mammosphere conditions for two days (2d) and three weeks (3w), using E and M-specific genes. Columns represent individual cells. Gene expression measured below background is shown in gray. (E) Mean expression per cell of 10 E-specific (*CDH1*, *CD24*, *EPCAM*, *IL1B*, *KRT5*, *LCN2*, *TP63*, *TRAIL*, *SLPI*, *S100A8*) and 7 M-specific genes (*ABCA6*, *DCN*, *IL1R1*, *PCOLCE*, *WNT5A*, *VIM*, *ZEB2*) in the E/M state space of individual cells grown in adhesion and three weeks as mammospheres (F). Crosses indicate mean expression of all 12 measured cells. P-values between the indicated groups of cells were determined using a cross-match test to discriminate by mean of E and M gene expression (S5 Table). Results with respect to statistically significant difference between gene expression in adherent E and M cells were observed in three independent experiments for single cells as well as well as for 100 cell pooled samples.

As expected, freshly FACS-sorted CD24+/CD44- cells from immune-stained HP cell populations (Fig 3A) had the typical E morphology (similar to HP cell cultures) whereas the CD24-/CD44+ cells had M morphology as observed by light microscopy (Fig 1A). The sorted subpopulations appeared to be stable as more than 88% of the cells remained within their respective CD24/CD44 quadrants after 10 days of re-culturing in adhesion and mostly resembled the single cell-derived E and M clones with respect to CD24/CD44 expression (Fig 3B and S1 Fig), but also both of them could slowly regenerate the opposite cell-type (Fig 3B).

In mammosphere assays not only cells from the E and M clones but also freshly sorted CD24+/CD44-(E) and CD24-/CD44+(M) cells could form sphere-like aggregates which were capable of survival and proliferation because carboxyfluorescein diacetate succinimidyl ester (CSFE)-labeled and sorted E and M cell-types both lost their CSFE label during mammosphere culture (S4A Fig). To compare E and M cells with respect to the stemness properties, plasticity and/or self-renewal, we analyzed single cell suspensions of dissociated mammospheres for CD24 and CD44 expression. Most cells of both freshly sorted CD24+/CD44-(E) cells and the E-clones had nearly completely converted to the CD24-/CD44+(M) state under mammosphere conditions (Fig 3C and S4 Fig), suggesting a high phenotypic plasticity of E cells. Conversely, M cells were significantly less plastic because neither freshly sorted CD24-/CD44+(M) cells nor cells from M clones had converted to the CD24+/CD44-(E) state in suspension mammosphere conditions (Fig 3C and S4 Fig). Consistent with the surprising low plasticity of CD24-/CD44+(M) cells in mammospheres, all five single cell-derived M clones lacked the capacity to generate a distinct CD24+/CD44-(E) population even when grown in adhesion for 5 passages (Fig 3B and S1 Fig) or 20 passages (data not shown). By contrast, in line with the high plasticity of E cells seen in mammosphere cultures, most E clones spontaneously re-established a small but stable CD24-/CD44+(M) subpopulation upon passaging in adhesion conditions within about 5 passages, establishing a heterogeneous culture that resembled the parental HP cell population as was previously reported for less transformed HMEC-derived HME cells [36].

Independent of the cell of origin (E or M clones, or freshly sorted E and M cells) the vast majority (65%-95%) of cells growing in suspension as mammospheres were CD24-/CD44+(M) cells (Fig 3C and S4 Fig), while cells in the CD24+/CD44-(E) state were usually not detected as has also been observed in primary breast tumor cells [42]. Thus, while the CD24+/CD44-(E) cells appeared highly plastic, the CD24-/CD44+(M) phenotype was not plastic, but by contrast had a proliferation advantage in suspension, indicating ‘self-renewal’ capacity with respect to its CD24/CD44 phenotype. Therefore, in the transition from adhesion to mammosphere culture, the stemness traits ‘plasticity’ and ‘self-renewal’ are uncoupled and segregate with either the CD24+/CD44-(E) or the CD24-/CD44+(M) phenotypes, respectively, challenging the widely held notion that the M phenotype but not the E phenotype embodies stemness.

### Single cell qPCR analysis reveals existence of CD44+ hybrid E/M cells in E and M cell-derived mammospheres

The enrichment for CD24-/CD44+ cells in mammospheres (Fig 3C) would suggest that the vast majority of cells in suspension cultures became ‘mesenchymal’ which is in conflict with the mammosphere transcriptome data (Fig 1D). This raised the possibility that cell-surface expression of CD24 and CD44 markers may not accurately reflect the E *versus* M duality. Since promiscuous gene expression within a cell is often a hallmark of stemness, we next considered the appearance ‘hybrid E/M cells’ not with respect to cell surface CD24 and CD44 expression but to a set of RNA-expression of E and M-specific genes. This could account for the increase of E gene expression in transcriptomes of the M clone-derived mammosphere that contained apparently homogenous CD24-/CD44 cells. Thus, we performed single cell qPCR analyses [47] to simultaneously measure the transcript levels of E- and M-specific genes in the E cell-lines (E5 and HP) and M cell-lines (M4 and M5 clones). We selected 10 E-specific and 11 M-specific genes from our differentially expressed predictive signatures (Fig 1, S1 and S2 Tables) that had a relatively high baseline expression to facilitate detection on single cell level but also included genes that were previously reported to be characteristic of E (*CDH1*, *EPCAM*,*CD24*) or M cells (*CDH2*, *FN1*, *VIM*, *CD44*) as well as other genes previously associated with stemness (S5 Fig and S4 Table).

As expected, in adherent cultures all individual E genes were higher expressed in individual E cell-types while most M genes were higher expressed in M cell-types (Fig 3D). Interestingly, some of previously reported genes to be M-specific were only occasionally (*FN1*, *CDH2*) or not at all (*CD44*, *SNAI1*) characteristic for M clones in whole transcriptomes of sorted small cell pools (100 cells) of adherent E versus M cells, nor did their expression show a larger than 2-fold difference between individual morphologically E and M cells (S5 Fig). Therefore, we focused on 7 M-specific genes (*ABCA6*, *DCN*, *IL1R1*, *PCOLCE*, *WNT5A*, *VIM*, *ZEB2*) most of which were also contained in the predictive HMLER_EM signatures (S1 and S2 Tables). Interestingly, the M genes *DCN*, *IL1R1*, *PCOLCE* and *VIM* were also expressed in some individual E cells. By contrast all ten E genes selected based on whole population transcriptomes (*CDH1*, *CD24*, *EPCAM*, *IL1B*, *KRT5*, *LCN2*, *TP63*, *TRAIL*, *SLPI*, *S100A8)* were exclusively expressed in E cells (with exception *KRT5* in 1 of 24 M cells) confirming their identity as E genes at the single cell level.

To assign the degree of E and M phenotype in individual cells, the transcript levels of all tested 10 E and 7 M-specific genes, measured by single-cell qPCR, were summarized by projecting each cell into a position in an E-M state space, spanned by the E and M axes which represent the aggregate normalized expression value of these E or M-specific genes, respectively (Fig 3E, see Experimental Procedures for details). In adhesion culture, E cells displayed substantial M-gene expression as manifest by their position closer to the diagonal: 22 of 24 E cells co-expressed E and M genes. This promiscuity of the nominal E cells was consistent with the high plasticity of E cells (Fig 3C). By contrast, 23 of 24 M cells exclusively expressed M-genes, which was on average 10-fold higher than in E cells. Therefore, M cells showed barely any overlap with HP or E5 cells in the E-M state space (p = 2.4*10^–5^, cross-match statistical test, see S5 Table and S1 Methods). Consistently, in analogy to their similar cellular morphology and CD24/CD44 expression in adhesion 12 HP and 12 E5 cells (p = 0.23) as well as the 12 M4 and the 12 M5 cells (p = 0.23) were not discriminable using the same markers. Thus our data confirmed good reproducibility of the cells’ state in the E-M state space for different HMLER cell lines, independent of clonal variations.

When grown as mammospheres, the average expression values (crosses in Fig 3E) of dissociated cells in all the analyzed cell-lines shifted toward the diagonal and showed similar expression patterns, suggesting a hybrid gene expression state in the E-M state space compared to the respective adherent E and M cell populations (Fig 3F). A correlation plot of all 46 analyzed genes in individual cells confirmed that mammospheres could indeed be seen as heterogeneous intermediate between the E and M state (S2D Fig). Specifically, in mammospheres we could distinguish two types of cells in the E-M state space: one type expressed only M genes, similar to adherent M clones; the other type on the diagonal showed the intermediate E/M transcript pattern. This hybrid E/M state was the collective result of individual cells in the mammospheres displaying ‘spotty’ expression of few individual markers of the opposite type (Fig 3D and 3F). The difference of gene expression pattern between the 24 adherent M cells and the 24 mammosphere-derived M cells was significant (p = 0.014) as was the difference between the 24 adherent E cells versus 24 mammosphere-derived E cells (p = 0.014). By contrast, within this same scheme of aggregating E and M gene expression value alone we could not discriminate between E- and M-derived cells anymore (p = 0.3) once the cells had grown as mammospheres (S5 Table), consistent with the convergence to the intermediate state we had observed in whole population arrays (Fig 1D and 1F). These data also demonstrated that some E cells could undergo complete EMT towards a state resembling differentiated M clones since they moved to the position of the ‘pure’ adherent M cells in the E-M state space, underscoring the plasticity of E5 and HP cells. Since M clones could express only one or two individual E genes in a few cells (Fig 3D) but they never converted to ‘pure’ E cells, this may suggest a proclivity to ‘incomplete’ or ‘partial’ MET during mammosphere formation.

In conclusion, single cell qPCR analysis and the projection into the E-M state space revealed the existence, in the aggregate, of hybrid E/M cells in mammospheres of all examined E and M cell-lines. Thus, the presence of hybrid E/M cells in mostly CD44+ cells-containing mammospheres explained why CD44 expression can both be associated with the EM state (in mammospheres) and with the M state (in adherent M clones).

### Co-culture of E and M cell-types synergistically increases mammosphere formation and self-renewal

The increased stemness and aggressiveness associated with co-expression of E and M signatures in luminal B and basal breast tumors (Fig 2) and of mammospheres derived from E and M cell-lines (Fig 1) can in principle result from one or both of two scenarios: (1) from a heterogeneity of cell composition, i.e., *mixture* of E and M cells, or (2) from the hybrid E/M cells (Fig 3F) due to co-expression of E and M genes within the same cell.

**Fig 4:**
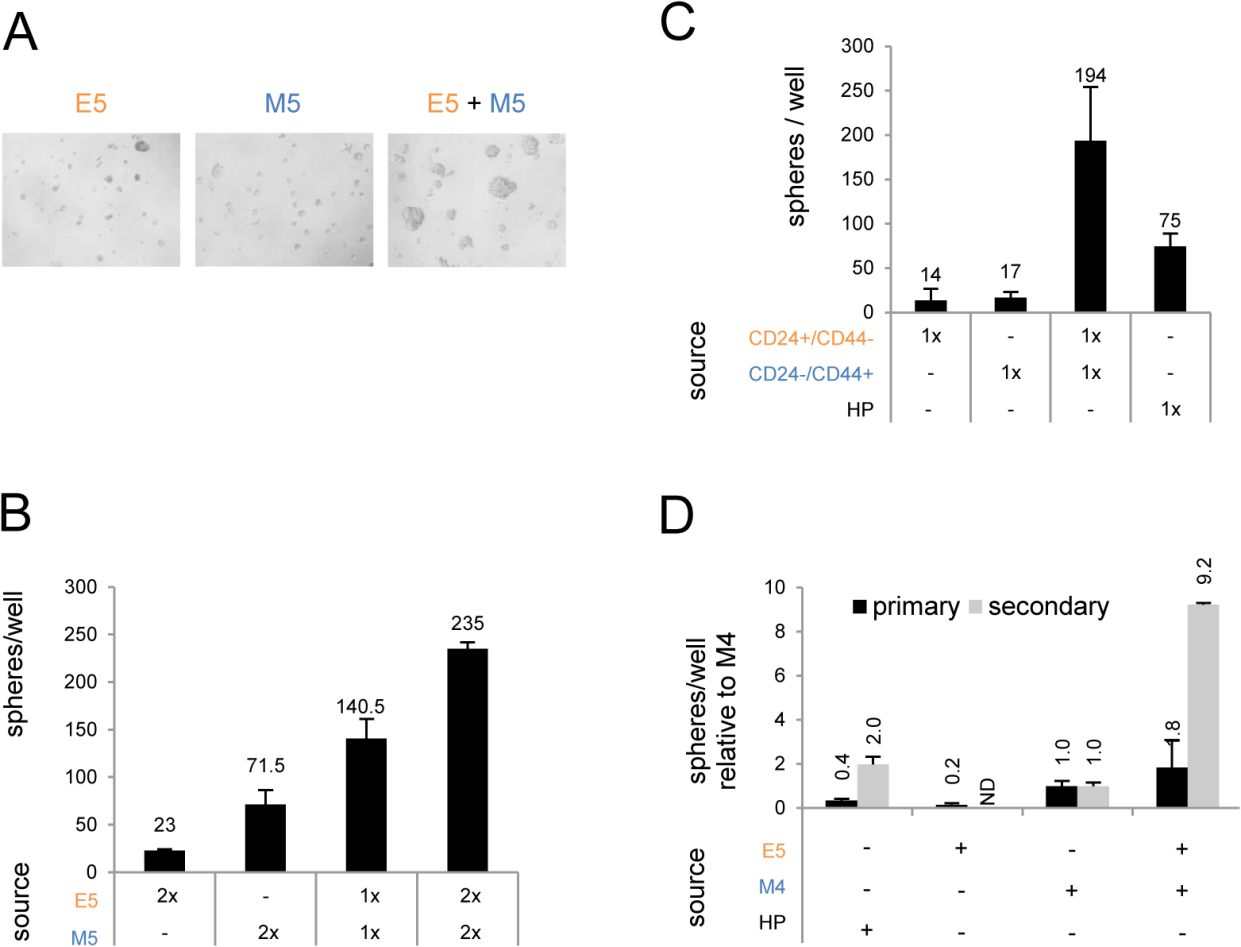
Co-culture of E and M cell-types synergistically increases mammosphere formation. (A) Representative light microscope images (10x objective) from mammospheres of 500 E5, 500 M5, and 500 E5 + 500 M5-derived mammospheres after two weeks suspension culture. (B) Total number of primary mammospheres derived from 500 (1x) or 1000 (2x) cells per cell-type seeded (from A) after two weeks suspension culture. (C) Total number of primary mammospheres grown from 500 (1x) freshly sorted CD24+/CD44-(E) cells, 500 CD24-/CD44+(M) cells, the co-culture, or 500 un-gated HP cells (‘all HP’) after two weeks of mammosphere formation. (D) Relative number of primary (seeded 2,000 cells per cell line per well) and secondary mammospheres from M4 grown with or without E5 cells or HP cells. After two weeks 10% of total number of dissociated cells from the indicated primary mammosphere samples were reseeded for secondary mammospheres and counted after one week. Mammospheres per well relative to that of M4-derived spheres are shown.

We first determined whether cooperation of E and M cells, the first scenario, *per se* can promote stemness which would explain its tight association with aggressiveness. Indeed, co-culturing of either E5 with M5 cells or of freshly sorted CD24+/CD44-(E) with CD24-/CD44+(M) cells substantially increased sizes (Fig 4A) and numbers (Fig 4B and 4C) of mammospheres compared to E cells or M cells cultured alone. In the mixed E+M mammosphere assays, mammosphere numbers were increased by two to six-fold relative to an expected additive effect. We observed this synergistic effect also when the total number of seeded cells was the same in the E+M co-culture as in the single cell-type cultures (Fig 4B). To test if co-presence of E and M cells in these mammospheres also resulted in increased self-renewal [11], we dissociated the mammospheres and generated ‘secondary’ mammosphere from 10% of the cells of the ‘primary’ mammosphere cultures. Indeed, the number of secondary mammospheres generated from cells derived from E5+M4-mammospheres was 10-fold larger compared to secondary mammospheres generated from cells derived from M4-mammospheres (Fig 4D). By contrast, mammospheres generated from E5 cells alone did not give rise to any secondary mammospheres. Thus, mixing populations of E+M cell-types can robustly increase mammosphere formation, indicative of cooperation between E and M cells that increases stemness trait of mammosphere formation and self-renewal. This suggested that E or M cells growing alone represent more ‘differentiated’ populations compared to E-M co-cultures which promote the ‘de-differentiation’ to a stem-like state.

Our observations of E-M cooperation are consistent with *in vivo* studies in which xenograft transplantation of a mixture of E and M cancer cells in mice produced more metastatic tumors than injection of pure cell populations alone [25,27] and with a recent study showing cooperating luminal and basal breast tumor subclones [48].

### Single CD24+/CD44+ cells are hybrid E/M cells and show increased stem-like properties

**Fig 5:**
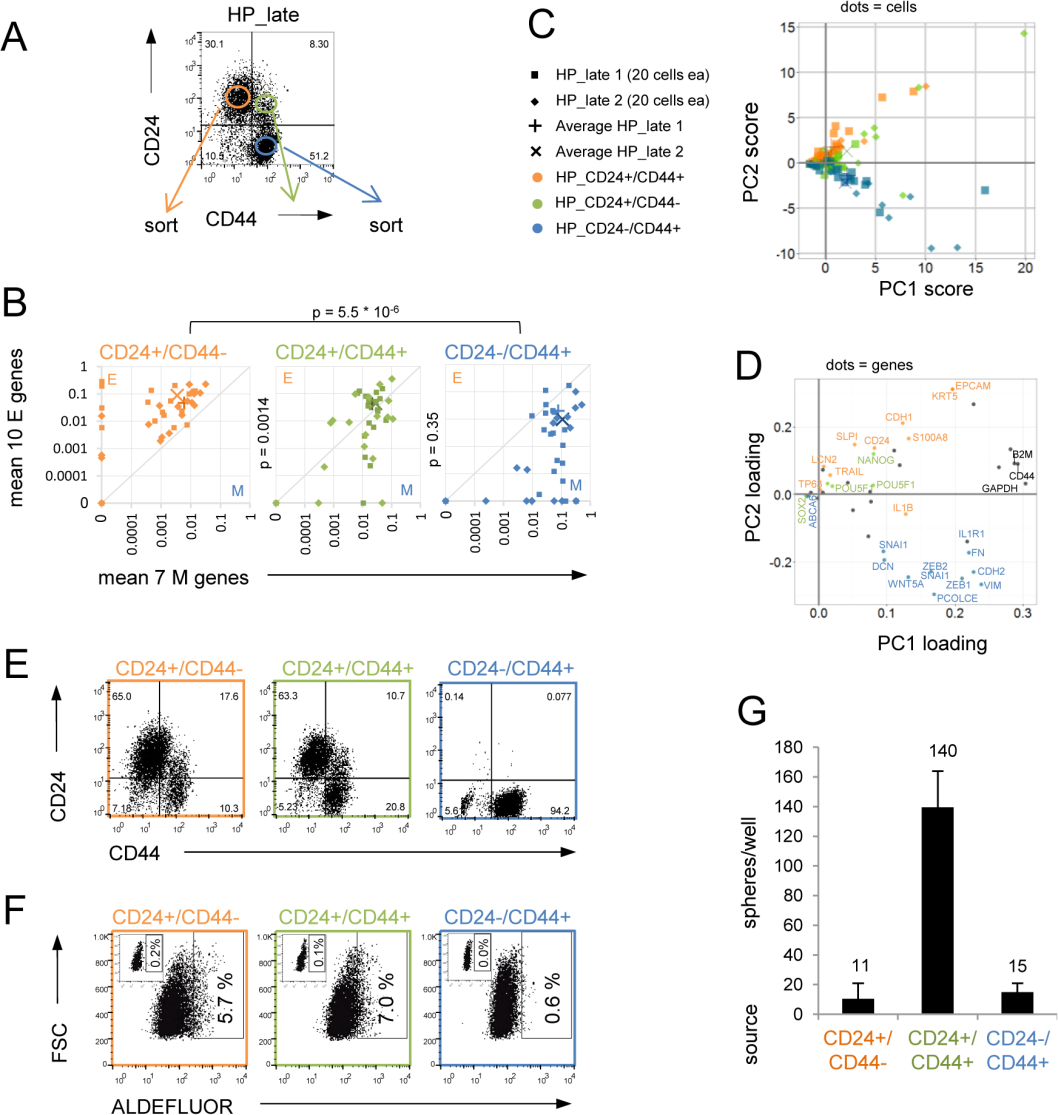
CD24+/CD44+ cells are hybrid E/M cells and have increased stem-like properties. (A) CD24/CD44 flow cytometry profile of HP_late cells. (B) Single cell qPCR analysis of 20 individual cells sorted from each of the six indicated CD24/CD44 subpopulations (from two independently passaged HP_late cell-lines). Mean expression of 10 E and 7 M genes (as in Fig 3) is visualized in the E-M state space. The crosses mark the average expression within the three different CD24/CD44 subpopulations (40 cells). Indicated p-values were determined using a cross-match test by comparing mean of E gene expression and/or M gene expression between indicated groups of single cells (S5 Table). (C) Principal component (PC) projections of the 120 individual cells as tested in (B) for the three different subpopulations colored according to their CD24/CD44 origin are shown. (D) PC loading projections of the 48 genes as tested in (B), showing the contribution of each gene to the first two PCs. E genes are colored in orange, M genes in blue, pluripotency genes in green, and housekeeping genes in black. (E) CD24/CD44 flow cytometry analysis of cells cultured under standard adhesion conditions for 10 days that had been freshly sorted from 100 cells of the three subpopulations from (A). (F) Assessment of ALDH1 activity in cultured cells derived from the three subpopulations (E) using the ALDEFLUOR assay. Gate shows percentage of ALDH1+ cells. Small insets show the negative control cells incubated with DEAB. (G) Mammosphere-forming ability per 1,000 sorted cells of the three HP_late derived cell populations (A) after two weeks in suspension.

To investigate whether the hybrid E/M state of the second scenario indeed represents increased stemness in HMLER cells, we took advantage of the fact that at later passages of HP cells (designated ‘HP_late’) with more balanced percentages of E and M cell populations showed a spontaneous distinct ‘double-positive’ CD24+/CD44+ subpopulation (Fig 5A, 30% CD24+/CD44-(E) cells, 51% CD24-/CD44+(M) cells, and 8% CD24+/CD44+ cells). These CD24+/CD44+ cells also sometimes spontaneously appeared in both E and M clones when passaged under adhesion conditions (S6 Fig) and thus their origin in the mixed HP populations remained unclear. We hypothesized that these CD24+/CD44+ cells represented an intermediate state between E and M cells, and thus, a possible subset of the hybrid E/M cells which could be functionally examined for stem-like properties by cell-sorting.

From each of the three FACS-sorted subpopulations (CD24+/CD44-, CD24+/CD44+ and CD24-/CD44+) from two independent HP_late cell-lines grown in adherence, we sorted 20 individual cells, using the FACS gates shown in Fig 5A, and analyzed 48 different transcripts using single-cell qPCR (S6 Fig). Examined genes included not only the E and M genes but also embryonic stem cell associated genes (*POU5F1*, *SOX2*, *NANOG*) as well as other genes expressed in breast cells (S4 Table, S6 Fig). First, we again projected the aggregate expression pattern of each cell into a position in the E-M state space (Fig 5B). Consistent with their expected E or M states, respectively, CD24+/CD44-(E) cells were in the upper left part of the E-M state space, and CD24-/CD44+(M) cells in the lower right part of the E-M state space with barely any overlap (1E cell in the M state and 3M cells in the E state space). Indeed, the CD24+/CD44+ double-positive cells occupied an intermediate state in the E-M state space along the diagonal, with about half of the cells in the E region, overlapping with CD24+/CD44-(E) cells and the other half in the M region, overlapping with CD24-/CD44+(M) cells. The difference between CD24+/CD44- and CD24-/CD44+ cells with respect to their position in the E-M state space was highly significant (p<10^–6^). Also, double positive cells were significantly different from CD24+/CD44-(E) cells (p = 10^–5^) but resembled more CD24-/CD44+(M) cells (p = 0.35 when using a cross-match test for both E and M genes, but p<0.004 using E or M gene sets alone in a Mann-Whitney U test, Fig 5B and S5 Table). By contrast, as expected, between the two independently passaged HP_late cell-lines CD24+/CD44-(E) cells, CD24+/CD44+(E/M), or CD24-/CD44+(M) cells were much more similar to cells with the same surface markers (p = 0.6, p = 0.9, p = 0.3,respectively, S5 Table), suggesting a good reproducibility of biological replicates for their positions in the E-M state space. Thus, the opposite expression patterns of CD24 and CD44 as the CD24+/CD44- and CD24-/CD44+ constellations in individual HP_late cells can accurately describe the E versus M dualism when both markers were simultaneously expressed in a population, and double positive cells could be discriminated to some degree as a distinct subpopulation from the CD24+/CD44- and CD24-/CD44+ cells.

Principal Component Analysis (PCA) of the 48-gene profiles of the individual cells confirmed the intermediate position of the CD24+/CD44+ cells population between the E and M cells with respect to the second component (PC2), thus confirming that individual CD24+/CD44+ cells were in a hybrid E/M state (Fig 5C). The nearly complete segregation of E genes and M genes in the second component confirmed our previous choice of E and M genes to discriminate between the most distinct cell-types in HMLER cells (Fig 5D and S6 Fig).

To compare the stemness capacities of these three cell-types we expanded the sorted populations for one week in adhesion culture. Both, the CD24+/CD44-(E) cells and CD24+/CD44+(E/M) cells were highly plastic, since they both gave rise to the more extreme E and M cells (Fig 5E). However, CD24+/CD44- cells were more stable (remaining 65% CD24+/CD44- cells) while only 11% CD24+/CD44+ cells persisted for 10 days in adhesion. Accordingly, ALDH1 activity, a marker for stemness in many tissues [49], was highest in the CD24+/CD44+(E/M) and CD24+/CD44-(E) cells (7.0% or 5.7%, respectively) (Fig 5F) while low (0.6%) in the CD24-/CD44+(M) cells which, as expected did not display phenotypic plasticity (Fig 5E). Thus, capacity to generate ALDH1+ progeny segregated with phenotypic plasticity. Remarkably, CD24+/CD44+(E/M) cells formed mammospheres with at least nine-fold higher efficiency than either sorted E or M cells alone (Fig 5G).

Taken together, these results show that CD24+/CD44+ double positive cells are (1) indeed hybrid E/M cells with respect to expression of multiple E and M genes and (2) exhibit stem-like character, including a substantially higher mammosphere forming capacity relative to the other more pure differentiated E and M populations, and more ALDH1+ progeny. Thus, CD44+ expression state can either be associated with stemness and the hybrid E/M state or with a more differentiated and more pure M state. Consistent with our data, in other tumor cell-lines derived from breast [50] and of other tissues [51,52] the CD24+/CD44+ cell subpopulation has previously been associated with a CSC-enriched phenotype due to increased tumorigenicity in mice.

## Discussion

Our results suggest that the co-expression of E and M markers in breast cancer at the whole-tumor level, producing an intermediate pattern between E and M, might scale with the stemness character, thereby accounting for its association with poor outcome (Fig 2). Here we show experimentally the two reasons that explain this relationship: First, the mixture of cells in the E and M states promotes stemness (measured as mammosphere formation) through the inter-cell cooperation between these two distinct cell-types (Fig 4); and second, the co-expression of E and M genes in the very same cell, which we call here hybrid E/M cells (Figs 3 and 5) also promotes stemness and mammosphere formation. While either one of these two scenarios in itself would result in the apparent intermediate E-M expression signature in the entire tumors, they are intertwined in that the former, cooperation, promoted the latter, stem-like hybrid cells (Figs 3 and 4). Of note, also *vice versa* some hybrid E/M cells (CD24+/CD44+) but not E clones (also individually co-expressing E and M genes, Fig 3E) could promote E-M cell cooperation in proliferating populations (Fig 5E and 5G), suggesting that not only RNA but also protein expression of both epithelial (e.g. CD24) and mesenchymal (e.g. CD44) states is necessary for mammosphere formation.

The interaction of both mechanisms for producing the intermediate E/M gene expression signature, cooperation and the presence of hybrid E/M cells may account for the robustness with which E/M co-expression predicts poor outcome in both ER+ and ER- breast cancer – to our knowledge the first gene expression signature that predicts outcome independent of tumor- subtype in breast cancer (Fig 2). Intriguingly, the predictive potential of our EM signature (suggestive of stemness) outperformed other signatures previously associated with poor outcome in breast cancer, such as signatures reflecting proliferation (PCNA-metagene and mammaprint) or those containing embryonic stem cell-associated transcription factors (NOS-TFs). These observations are consistent with the recent findings that alternative lineage specifiers can replace OCT4 and SOX2 in the reprogramming of human fibroblasts [53] confirming they can be encoded in a distributed manner –through promiscuous expression patterns.

**Fig 6:**
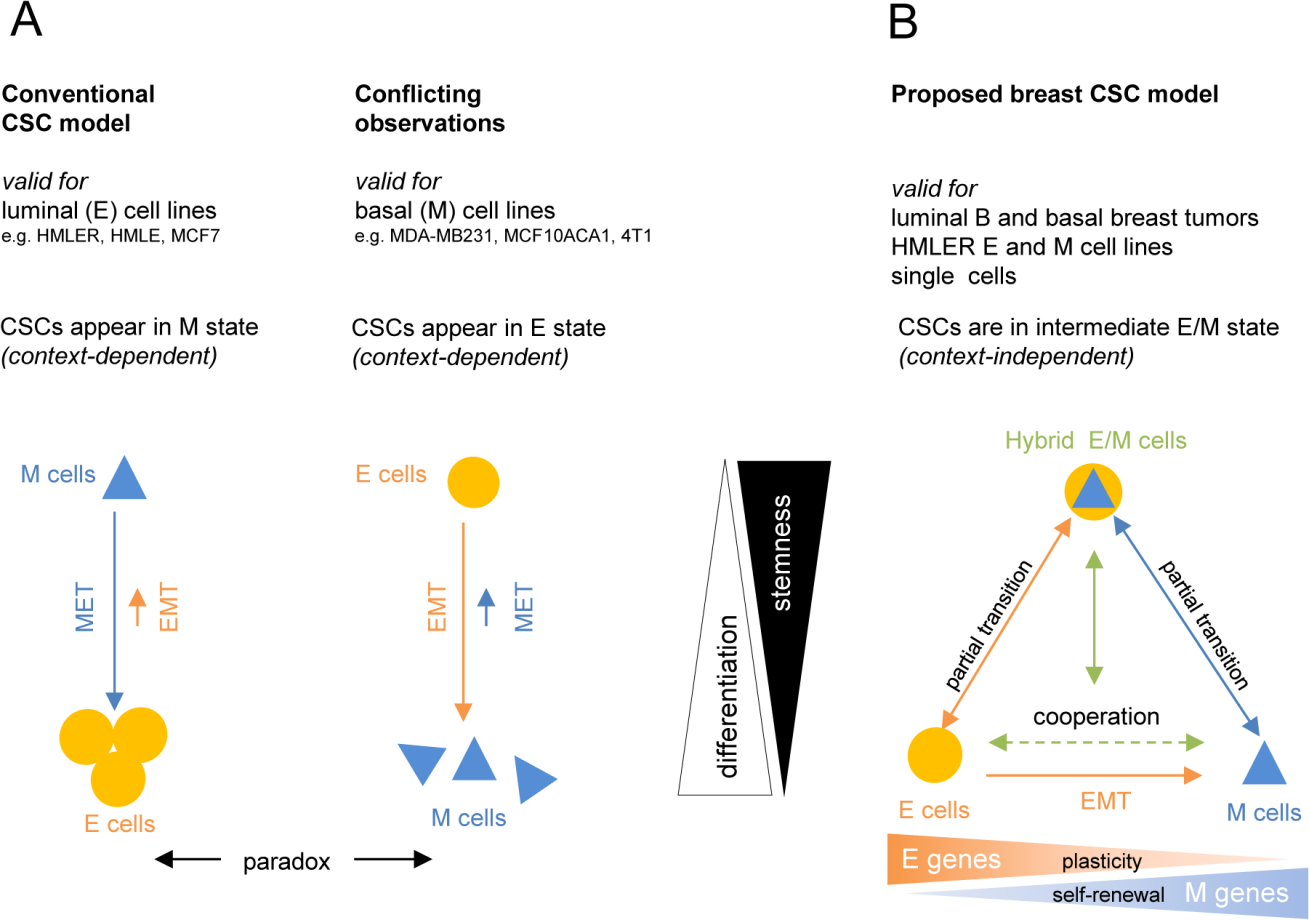
The conventional versus a new integrative CSC model for breast cancer. (A) In luminal epithelial cell-lines (e.g. in HMLE, HMLER, MCF7) the conventional model describes breast CSCs as M cells and ‘non cancer stem cells’ as E cells. Paradoxically, in more basal mesenchymal cancer cell-lines (MDA-MB231 cells, MCF10ACA1, 4T1) the more E gene-expressing cells are associated with CSC-properties. Thus, the identity of CSCs appears to be context-dependent. (B) Our model for CSCs integrates and explains these paradoxes by the existence of stem-like intermediate hybrid E/M cells independent of the tumor cell line. We propose that compared to more ‘polarized’ differentiated E (capable of plasticity) and M cell-types (capable of self-renewal), undifferentiated hybrid E/M cells can generate more mammospheres and heterogeneous progeny due to their capacity of both, self-renewal and plasticity. Arrows indicate possible state transitions (incomplete EMT and MET) between the instable hybrid E/M state and the extreme stable E and M states. Stemness of the intermediate E/M state is reflected both by presence of stem-like hybrid E/M cells and by co-presence of E and M cell-types due to cooperation. Context-independency of the stemness of the intermediate E/M state explains the paradoxical context-dependent meaning of E genes in basal tumors and M cell lines and stemness of M genes in luminal B tumors and E cell-lines (A).

Our data provide an explanation for a series of paradoxical reports on the role of E and M gene expression in CSCs *in vitro* and *in vivo* (Fig 6A): in luminal epithelial cell lines, stemness and CSC have been linked to expression of M-specific genes [15]; conversely, in basal mesenchymal cell lines stemness has been associated with expression of E genes [20,23–25]. Our results support a new testable breast CSC model (Fig 6B) that is consistent with our hypothesis in that the postulated small subpopulation of CSCs resides in the intermediate hybrid E/M state, independent of the tumor and cell-line context (luminal epithelial and basal mesenchymal breast cell lines and luminal ER+ and basal ER- tumors): stem-like hybrid E/M cells can arise in both the E and M cell populations through incomplete EMT and MET, respectively. This departs from the paradigm in which CSC are the result of the process of EMT [13,14].

Our CSC model, experimentally supported and consistent with clinical data, is in line with previous theoretical work that postulate an intermediate E/M state [5,28,29,54–56] and its association with stemness. These studies suggested that the hybrid E/M state arises in a dynamical fashion as a (meta)stable attractor state in a gene regulatory circuit composed of mutually inhibitory circuits of miR-34/SNAI1 [54,56], miR-200/ZEB [28,56], or LIN28/let7 [55], with intermediate levels of both miR200 and ZEB or LIN28 and let7 characterizing the intermediate stem-like hybrid E/M state, similar to the negative feedback loops seen in other decisions at branch points in cell development [57]. Furthermore, the LIN-28/let-7 circuit connects the intermediate hybrid E/M state with expression of *intermediate*, stemness-associated levels of the pluripotency factor POU5F1/OCT4 [58]. However these models do not consider cell population heterogeneity and ensuing heterotypic E-M cooperation which introduces a non-cell autonomous element in cell-state dynamics which, as we have shown, affords additional stability to the hybrid state. Given the key role of miRNAs in these models, extracellular presence of mir-200 or let7 could be a key component of this cooperation.

We found in our *in vitro* model that M clones were irreversibly locked in a more mesenchymal and less plastic state compared to E-cell-types both in adhesion (S1A Fig) or suspension cultures (Fig 3 and S4B Fig). This is supported by reports that TGFβ-treated MCF10A cells undergo an irreversible switch to the M-state regulated by the miR-200/ZEB circuit [29,56]. By contrast, MET has been observed during reprogramming of fibroblasts [59,60] but in the absence of single cell resolution data using appropriate markers it remains unclear whether MET was partial or complete in these studies. However, a hidden potential to undergo MET may depend on the respective cells, their underlying epigenetic state and could be unleashed in few individual cells by genetic manipulation with inducing factors, such as transduction with vectors encoding pluripotency factors [61] and subsequent selection. In accordance with our model, the irreversibility of the pure M state after complete EMT as well as stemness of the intermediate state was also suggested in a very similar, independently developed ‘gradient EMT model’ which also assumes that cells that had differentiated to the mesenchymal state are more committed and thus less plastic than E cells or hybrid E/M cells [62].

Our experimental data suggest that compared to the hybrid E/M state the more differentiated E and M cells still exhibit aspects of stemness which are distinct in these two cell-types: we found that cells expressing E genes exhibited phenotypic plasticity while cells expressing M genes displayed self-renewal in suspension to cells (Fig 3). Expression of either set of genes alone correlated with less mammosphere formation capacity than co-expression of E and M genes. This confirms that the “extreme” E and M states represent two distinct “differentiation states” relative to the stem-like intermediate E/M state. The complementarity of plasticity and self-renewal (both of which are necessary characteristics of stemness) may underlie the synergism between E and M cells in mammosphere formation (Fig 4). It is likely that complex and possibly highly redundant mechanisms involving cell-cell communication play a role in the cooperation between E and M cells due to the redundancy in EM signatures to predict poor outcome (Fig 2 and S3 Fig). The central role of such cooperation is manifest through complementary expression of E and M cytokine and their cognate receptor pairs on E and M cells (or *vice versa*), such as IL1B (E cells) and IL1R1 (M cells) (S5B Fig) and in many other heterotypic E and M cell interactions relevant during embryogenesis [63]. Complicating the simple notion of a symmetric dualism of the E and M type cells, we note that despite the obvious distinction between these cell states in terms of morphology (which was strongly correlated with the mirror CD24+/CD44- and CD24-/CD44+ surface marker status), analysis of single cell transcript patterns suggests that the majority of E cells also express components of the M cell program (Fig 3E), in line with their higher plasticity compared to M cells (Fig 3B and 3C).

A recent study on primary tumors and tumor cell lines demonstrated that CSCs are either generated by EMT or MET [34] which is consistent with our experimental data comparing adherent cells *versus* mammospheres which could be generated by EMT or MET (Fig 1D–1F). However, Liu *et al* also demonstrated that the CSCs characterized either by expression of the mesenchymal marker CD44 or by the epithelial and stemness marker ALDH1 can exist in two distinct gene expression states overlapping in only a few expressed genes. This seems contradictory to our finding of a hybrid E/M stem-like state that can arise from E or M state. However, since ALDH1+ and CD44+ cell populations are only *enriched* for CSCs but do not represent pure CSC populations and thus also contain bulk non-CSCs, the non-overlapping portion of the gene expression profiles of these two populations could be explained by the bulk non-CSC.

In conclusion, our work suggests that co-expression of the E and M markers in tumors is the manifestation of both cellular heterogeneity due to co-presence of cooperating E and M cells, as well as co-expression of E and M-specific genes in the stem-like hybrid E/M cells. Although the experiments were performed solely in HMLER cells, chosen for their intriguing and well-trackable dynamics between the E and M states within a single cell line, our findings erect a general principle for the relationship between E and M-state that for the first time explains observations in cancer-derived cell lines, in animal models as well as tumor transcriptomes of breast cancer patients.

*In vivo*, variations in populations of circulating tumor cells (CTCs) ranging from E over hybrid E/M to M states have been observed and are found to form in highly tumorigenic [64] cell clusters of mixed cell-types [65]. In addition, human breast tumor-derived CTCs co-expressing E and M protein markers, such as EPCAM and CD44 have been shown to initiate metastases in mice [66]. Together with our data these observations suggest that co-expression of E and M signatures can serve both as markers for aggressiveness in whole breast tumors as well as in individual CTCs. Finally, since the metastatic ability appears to be an inherent property of all breast tumor subtypes [45], and is linked to the apparent intermediate E/M signatures, on can envision that dual therapeutic targeting of both the E and M markers represents the targeting of both key elements, hybrid E/M cells and the E-M cell cooperation which may improve survival of all cancer patients independent of breast tumor-subtypes.

## Experimental Procedures

### Cell culture

The mammary epithelial cell (HMEC)-derived HMLER cells were kindly provided by Robert A. Weinberg (Whitehead Institute). ‘HP’ cells are defined here as a parental HMLER population that ranged between 5% and 30% CD24-/CD44+(M) cells, while later passages with >30% M cells were designated ‘HP_late’ cells. HP cells and single cell-derived E and M clones were cultured under serum-free standard cell conditions in a 1:1 mix DMEM (Life Technologies)/MEGM (Lonza). Cells were passages twice a week using trypsin which got inactivated by washing with 10% FBS. Mammospheres were generated as described [11]. Briefly, cells were enzymatically (30min trypsin) and mechanically detached and dissociated, and between 2,500 to 10,000 cells/ml (clonogenic densities, except Fig 4D) were either sorted or seeded into ultra-low attachment plates (Corning) and cultured for 2 to 3 weeks in DMEM/MEGM supplemented with 1x B27 (Life Technologies) and 1% methylcellulose (viscosity 4,000cP, Sigma).

### Imaging

Cells were photographed on a Leica DM IL LED instrument (Leica) with a DFC345 FX camera or on a DeltaVision Core (GE Healthcare).

### Gene expression arrays and gene sets

RNA of adherent growing cells was isolated by lysing adherent PBS-washed cells by addition of TRIzol (Life Technologies) in the dish and purified according to the manufacturer’s instructions. Suspension cultures were washed with PBS and pellets were lysed by addition of TRIzol and rigorous pipetting. RNA was purified by standard ethanol precipitation followed by additional purification using the RNeasy micro kit (Qiagen). Integrity of RNA was verified by a 2100 Bioanalyzer instrument (Agilent), and only RNA with a RIN >8 was used for subsequent microarray analyses. 50 ng to 100 ng RNA was labeled using the one color Low Input Quick Amp Labeling Kit (Agilent), and labeled probes were run on Human 4x44K Microarrays (Agilent) according to the manufacturer’s instructions. Spot quantitation was performed using Agilent’s Feature Extractor software and all data were then entered into a custom-designed database, SLIMarray (http://slimarray.systemsbiology.net), and then uploaded into Genedata Analyst 7.0 (Genedata, Basel, Switzerland). Data normalization was performed in Genedata Analyst using central tendency followed by relative normalization. Transcripts showing differential expression (p value < 0.01) between two different morphological phenotypes (E clones E1, E2, E3, E4, E5, E6 and M clones M2, M3, M4, M5) were identified by Limma and ranked according to their ‘effect size‘. The complete microarray dataset has been deposited in NCBI’s Gene Expression Omnibus and is accessible through GEO accession number GSE66527.

### Patient classification and Kaplan-Meier survival analysis

Using the ‘Kaplan-Meier Plotter’ online tool (http://kmplot.com/analysis/) and the 2014 dataset comprising up to 3458 total patients (1115 patients for ‘overall survival data’), we subdivided patients into four clinically relevant intrinsic subtypes based on the St. Gallen criteria using bimodal expression of estrogen receptor (ER, *ESR*), expression of HER2 (*HER2*), and the proliferation marker Ki67 (*MKI67*) into luminal A (*ESR+*, *HER-*, *MKI67* ^low^), luminal B (*ESR+*, *HER2-*, *MKI67* ^high^ and *ESR+*, *HER2+*), basal (*ESR-*, *HER2-*) and HER2+ (*ESR-*, *HER2+*) patients [43]. The following probes and cutoffs were used [67]: ESR probe 205225_at (cutoff 500), MKI67 probe 212021_s_at (cutoff: 470), and HER2 probe 216836_s_at (cutoff: 4800).

Hazard ratio (‘HR’, within 95% confidence intervals) and logrank p-values (Cox’ proportional hazard ratio analysis) for overall survival of breast cancer patients [43] was determined by using the mean expression of the indicated E and M signatures (for gene lists see S2 Table). We chose the ‘autoselect best cutoff’ option for subdividing gene expression in primary tumors by median, quartile or tertile expression, and ‘JetSet best probe set’ [68] options to assess the hazard ratio and logrank p-value for overall or relapse-free survival.

### Immunofluorescent staining and flow cytometry analysis

Adherent cells or mammospheres were trypsinized and mechanically disrupted to generate single cell suspensions. For CD24/CD44 profiling cells were stained with antibodies (αCD24-PE: clone ML5, αCD44-FITC or αCD44-APC: clone G44-26, BD-Biosciences), that were diluted 1:25 in DMEM supplemented with 2% FBS. To discriminate apoptotic cells before sorting for qPCR analyses, cells were incubated with AnnexinV/FITC and propidium iodide (PI) (apoptosis detection kit, BioVision). For detection of active ALDH1 single cell suspensions were subjected to the ALDEFLUOR assay (Stem Cell Technologies) with diethylaminobenzaldehyde (DEAB)-treated cells as control, as recommended by the manufacturer. Flow cytometry data acquisition was performed on a FACSAria II SORP (Becton Dickinson) and analyzed with FlowJo software (Tree Star, vX.0.6). To determine the CD24/CD44 quadrants in the clones and mammosphere derived cell populations we simultaneously stained parental HMLER cells as a reference to discriminate between the opposite CD24+/CD44- and CD24-/CD44+ populations, and used the same gates for all cells stained and measured at the same time. All data shown are representative of at least two experiments performed in duplicates.

### Cell sorting and single cell qPCR analyses

Only singlet cells of equal FSC/SSC morphologies within the middle of the respective fluorescent gates were sorted. Cells were sorted directly into 96 well-plates and cultured in suspension or adhesion conditions. For qRT-PCR analysis of single and 100 AnnexinV-/PI- (= live) cells were sorted into wells containing Taqman primers and Cells Direct One-step RT-PCR and pre-amplification mix (Life technologies) according to the Fluidigm single cell protocol using 18 cycles of preamplification. Gene expression was analyzed on a BioMark instrument (BioMark HD system, Fluidigm) using 48.48 Dynamic Array IFC chips (Fluidigm).

### Single cell data processing

Measured C_q_ (Ct) values were transformed into linear expression values according to Fluidigm protocol (Single cell application analysis): expression value = 2^Log2Ex^ with Log2Ex = C_bg_ – C_q_ with estimated background C_bg_ from reported C_q_ values of no template control (NTC) reactions. Reported maxima were verified not to be outliers. Non-detected data (C_q_≧C_bg_) were set to 0. Expression data was normalized to the maximum measured for the same gene across all single-cell samples. To plot single cell gene expression values into the E-M state space (Figs 3E, 3F and 5B), normalized expression values per single cell were averaged for all 10 E genes (‘mean E genes’) or 7 M genes (‘mean M genes’) and displayed as ‘aggregate’. For PCA analyses (Fig 5C and 5D) we used a total of 267 single cells which contained the three different subpopulations (HP_late 1 and HP_late 2) and about equal amounts of E and M cell-types (including clones, not shown). Each gene expression was centered to have a mean of zero and scaled to have a variance of 1. We then used the R function PRCOMP to determine PC scores and PC loading for the three subpopulations in HP_late populations. Raw Ct values for single cell data shown in Fig 3 are shown in S6 Table and for Fig 5 in S7 Table.

## Supporting Information

S1 Methods (DOCX)

S1 Fig (Related to Fig 1): Generation of epithelial (E) and mesenchymal (M) single cell-derived clones from HMLER cells. (A) Single cell sorting of parental HMLER cells generated morphologically stable E and M clones. Cells were expanded in adhesion under normal culture conditions. Characteristic CD24/CD44 FACS profiles of clones after about 5–10 passages are shown. Note the small but distinct CD24-/CD44+ (M) populations appearing in all E clones (circled), and the absence of CD24+/CD44- (E) cells in M clones. (B) Comparative expression of E, M, and EM gene sets (S2 Table) relative to basal B or luminal cancer cell lines with expression as assessed by the GOBO online tool (http://co.bmc.lu.se/gobo/) [39]. This classifies E genes as rather luminal-specific and M genes as more basal B-specific genes, and the EM gene set as neutral. The same relative association with E and luminal and M genes and basal cell lines, respectively, was found for the E_HMLER(150) and M_HMLER(150) signatures, as well as for E and M specific gene sets (data not shown) from Taube *et al* (S2 Table).
(PDF)

S2 Fig (Related to Fig 1): Dynamic expression of E and M genes in HMLER cell populations. (A,B) Heat maps of gene expression microarray data from adherent cells (adh) and suspended mammospheres (ms) shown after relative normalization towards the intermediate expression in E and M clones. (A) Expression of all transcripts that were enriched >5-fold in suspension grown mammospheres versus the respective adherent cultures. The data show that M genes (expressed higher in adherent M clones) are highly increased in HP mammospheres while E genes (higher expressed in adherent E clones) are increased in M4 mammospheres. Genes that are only enriched in HP mammospheres or only in M4 mammospheres were designated “context-dependent” and the intersection of genes enriched in both cell types as “context-independent transcripts”. (B) Expression of genes expected to be expressed and previously associated with high or low expression in HMEC-derived mammospheres [11]. (C) Pearson correlation for E_HMLER (150) and M_HMLER (150) gene expression in whole population microarrays of HP and M4 cells in adhesion and suspension. (D) Pearson correlation of single cell transcriptomes (Biomark, Fluidigm) measured for 12 cells per group (groups as in C, every column and line per individual cell) and 46 different genes (including, housekeeping genes, E and M-specific genes, pluripotency factor encoding genes, suspension-specific genes) as indicated in S4 Table. Data are from same experiment as shown in Fig 3D. Note the similarity of gene expression correlation in whole population arrays (C) and single cell qPCR analysis: in adhesion we observe a positive correlation between E cells, or between M cells, and anti-correlation between E and M cells, while mammosphere derived cells individual E and M cells cannot be discriminated anymore by correlation.
(PDF)

S3 Fig (Related to Fig 2): EM signatures predict poor outcome in ER+ and ER- breast cancer subtypes. Kaplan-Meier analysis for 6 different EM gene sets (24 genes) from HMLER and HMLE cell lines (S2 Table) in luminal B (epithelial) and basal (mesenchymal) breast cancer patients (Kaplan-Meier-Plotter data set 2010, 72 basal patients, 208 luminal B patients). Numbers of signatures with significant HRs indicating poor prognosis are indicated. Every triangle represents a different gene set. (A) Hazard ratio (HR) for overall survival is plotted against corresponding logrank p-values. HR of >1 indicates poor outcome, HR <1 indicates good outcome. (B) Hazard ratio for relapse-free survival is plotted against corresponding logrank p-values. (C) Hazard ratios for overall survival is plotted against corresponding permuted p-values. Permuted p-values were determined from a permutation test of 10^6^ samples for the respective signature by calculating the probability of achieving a more extreme result with a random gene set of the same size for overall survival of patients. (D) Kaplan–Meier analyses for overall patient survival with gene sets derived by bootstrapping from all 22,772 analyzed probes. 10,000 different gene sets consisting of 10 randomly picked genes were analyzed (every dot represents a different gene set). (E) Kaplan–Meier analyses for overall patient survival with gene sets derived by bootstrapping from the most extreme 24 EM_HMLER genes. 10,000 different gene sets consisting of 10 randomly picked genes were analyzed.
(PDF)

S4 Fig (Related to Fig 3): CD24+/CD44- (E) cells can proliferate under suspension mammospheres conditions and are not generated by CD24-/CD44+ (M) cells. (A) 500 CSFE-positive (CSFE+) cells expressing CD24+ /CD44- (E_CSFE) cells or CD24-/CD44+ M cells (M_CSFE) or 500 CSFE-negative (CSFE-, unlabeled) E and M cells (E_u, M_u) were sorted from HP cells as the indicated biological replicates and cultured in suspension mammosphere conditions for 2 weeks, harvested, and analyzed for their retained CSFE label by quantitative flow cytometry analyses. Bar graphs of relative numbers of CSFE- (proliferating, number indicates CSFE- cell numbers) and CSFE+ (label retaining non-proliferating) cells from dissociated mammospheres per well are shown. Note that the majority of replicates from both E and M cells lost their CSFE label, indicative of proliferation and survival, even though CSFE labeling also appeared toxic to E cells resulting in reduced cell numbers relative to unstained cells from the same source (compare E_CSFE and E_u). (B) Representative CD24/CD44 flow cytometry profiles of dissociated mammospheres (grown 2–3 weeks) from HMLER clones. Note that only cell lines that originally contained E cells (E5 and HP cells) still contained distinct CD24+/CD44- populations but M1 and M4 cells had only a spotty appearance of less than 1% CD24+/CD44- cells, supporting the plasticity of E cells and non-plasticity of M cells.
(PDF)

S5 Fig (Related to Fig 3): Expression of selected E and M genes in HMLER cell lines and HP subpopulations. (A) Heat map of selected E and M genes in microarrays of adherent HMLER-derived cell lines (HP cells, 3 different E clones, 3 different M clones with different passage numbers). The same microarray data as used for Fig 1A are shown as relative expression. (B) Heat map of relative gene expression of selected E and M genes assessed by qPCR analysis of 100 AnnexinV-/PI- (live) sorted cells of the indicated sources grown in adhesion (biological triplicates). Using these data we picked E and M genes for single cell qPCR analysis shown in Fig 3D–3F. Note that the usually with the M phenotype associated genes *SNAI1*, *FN*, *CDH2*, and *CD44* are not consistently differentially expressed between adherent morphological E and M cell types in arrays and/or qPCR analysis and thus were not used as M-specific genes. (C) Heat map of gene expression in single cells (before subtraction of background) from cell populations grown under adherent or mammosphere suspension conditions as determined by single cell qPCR analysis (same experiment as shown in Fig 3). No-template-controls (empty wells) are designated NTC. Not detected data points are shown in gray. Biclustering based on correlation analysis (using the heatmap.2 R function) of raw expression values including background of adherent and suspended E and M cells for the indicated genes clustered E and M genes into two separate large clusters (the ones used in qPCR analysis are circled in orange and blue), confirming their association with the opposite E and M cell-states. (D) Normalized average expression of individual genes in 24 adherent E cells (HP and E5 clone) versus in 24 adherent M cells (M4 and M5 clones). ‘Not qualifying M genes’ did neither show a biological significant effect-size in arrays, 100 cell- or single cell qPCR analyses. Asterisks mark genes statistically not significant to discriminate between E *versus* M state. Remarkably, possibly due to the spotty expression of both E and M genes at the single cell level (gray: not detected or high background as for *CDH1*, see C) a statistically significant difference of expression often did not coincide with a large effect-size between E and M cell types (e.g. *CDH1*, *TP63*, *ZEB2* or *SNAI1*), while *vice versa* a small effect size could correlate with a high statistically significant difference (*CDH2*, *FN1*, *IL1R1*, *VIM*).
(PDF)

S6 Fig (Related to Fig 5): CD24+/CD44+ cells can spontaneously arise from E and M clones. (A) Flow cytometry profile for cell surface CD24 and CD44 expression in HMLER clones E5 and M4 grown in adhesion. Quadrant gate coordinates were determined using simultaneously measured HP cells. (B) Single cell qPCR analysis for two independently passaged HP_late subpopulations (HP_late 1 and HP_late 2). Cells were stained for CD24 and CD44 and 20 cells per indicated cell line and gate were sorted. (C) PC1 and PC2 values of the individual cells (from Fig 5B and 5C) plotted against the variance of expression values per cell (expression value normalized to the maximum expression per gene in whole experiment). Cell subpopulations are labelled according to their CD24/CD44 marker expression during sorting (B). PC1 can be regarded as discriminating between the variance relative to the maximum (diversity), while PC2 was discriminating between E and M cell-types.
(PDF)

S1 Table (Related to Fig 1): E_HMLER (150) and M_HMLER (150) signatures. The 150 most differentially expressed HMLER genes associated with respective E or M morphology were derived from relative expression of grouped stable morphological E versus morphological M clones, respective fold expression between grouped E and M clones is shown in brackets (Limma, adjusted p-value < 0.01).
(XLSX)

S2 Table (Related to Fig 2): Signatures used for Kaplan-Meier analyses. EM gene sets were derived from HMLER (S1 Table) or from published gene sets derived from parental epithelial versus mesenchymal HMLE cells [22] or lung cancer mesenchymal versus lung cancer epithelial cell lines [38]. For HMLE gene sets mesenchymal HMLE cells had been generated by transduction with vectors encoding siCDH1, TWIST, GSC, SNAI1 or by treatment with TGFβ and E_HMLE genes were originally named EMT-DOWNREGULATED and M_HMLE genes were originally designated as EMT_UPREGULATED. For the lung E and M gene sets genes had been derived from microarrays from 93 lung cancer cell lines by comparing groups of cells with high VIM and low CDH1 (mesenchymal genes) and low VIM and high CDH1 (epithelial genes). Our EM gene sets were composed from the respective 30 or 12 most extremely expressed E genes and 30 or 12 M genes, to derive the EM signatures of 60 genes or 24 genes. The PCNA-metagene was derived by correlation expression analysis with expression of PCNA from >1200 breast cancer microarrays using the GOBO online tool (http://co.bmc.lu.se/gobo/)[39], while mammaprint [38] and NOS-TFs (targets of NANOG, OCT4 and SOX2 that are transcription factors themselves)[46] were derived from the literature.
(XLSX)

S3 Table (Related to Fig 1): Gene set enrichment analysis for mammospheres. GSEA using the indicated E and M gene sets was performed using standard GSEA analysis. Inverse regulation of E genes versus M genes from adhesion to suspension mammosphere cultures was observed, independent whether gene sets were derived from HMLE or HMLER cell lines.
(PDF)

S4 Table (Related to Figs 3D–3F and 5B): Taqman primer list. List of the Taqman assays used for single cell and 100 cell qPCR analyses shown in Figs 3 and 5 are indicated and their order numbers (Life Technologies) are shown.
(XLSX)

S5 Table (Related to Figs 3D–3F and 5B): Statistical analysis for single cell qPCR data. Normalized expression of the average expression of 10 E genes (*CDH1*, *CD24*, *EPCAM*, *IL1B*, *KRT5*, *LCN2*, *TP63*, *TRAIL*, *SLPI*, *S100A8*) and 7 M genes (*ABCA6*, *DCN*, *IL1R1*, *PCOLCE*, *WNT5A*, *VIM*, *ZEB2*) per cell was determined as plotted in the EM state space shown in Fig 3E and 3F for the indicated different cell populations all grown in different wells under either adhesion or suspension mammosphere conditions. To examine whether the difference as displayed in the EM state space was statistically significant between the different populations we used the cross-match test (for E and M gene expression) and Mann-Whitney U test for E gene expression or M gene expression. For the single cell data as displayed in Fig 5B, expression data from three different CD24/CD44 cell populations of single cells from adherent HP_late1 and HP_late 2 (plotted in the EM state space shown in Fig 5B) were tested for their differences using the cross-match test and Mann-Whitney U test.
(PDF)

S6 Table (Related to Fig 3D–3F): Raw single cell data. Raw measured Ct (Cq) values (48:48 chips, BioMark, Fluidigm) of single (1) and 100 cell qPCR data from HMLER-derived cell lines either cultured in adhesion (adh), under mammosphere suspension conditions for two days (2d) or for three weeks (3w). Primers for indicated genes were used as indicated in S4 Table. Empty fields represent no amplification or data with insufficient quality. No template control (NTC).
(XLSX)

S7 Table (Related to Fig 5B): Raw single cell data. Raw measured Ct values of single (1) and 100 cell qPCR data from HMLER-derived cell lines and different CD24/CD44 subpopulations cultured in adhesion conditions. Otherwise as for S6 Table.
(XLSX)
