# Supplementary material for: Stemness of the hybrid Epithelial/Mesenchymal State in Breast Cancer and Its Association with Poor Survival
Source: PLoS One. 2015 May 28;10(5):e0126522. doi: 10.1371/journal.pone.0126522 (PMC4447403; doi:10.1371/journal.pone.0126522)
Supplement: S1 Methods — (DOCX) [file pone.0126522.s001.docx]

# S1 Methods

## Kaplan-Meier survival analysis

Using the same parameters Kaplan Meier online tool but our own analysis tools (R programming Language using Bioconductor package, patient data set 2010, 1783 total patients with ‘relapse-free survival data’, 593 patients with ‘overall survival data’ (Györffy et al., 2010)) the HRs and logrank p-values were assessed for the gene sets shown in Table S2. Since the Kaplan-Meier-online tool offered downloading of the 2010 version of the patient database which only allowed simultaneously testing of about 24 different genes, for the following significance testing against random gene sets we did not use 60 but the smaller 24-genes comprising gene sets shown in S2 Table. To test the statistical significance how well the selected gene-sets stratify patients compared to random sets we performed a permutation test by sampling 10^6^ different gene sets consisting of 24 randomly chosen genes, and calculated hazard ratios based on the annotated patient cohorts. Reported p-values (termed ‘permuted p-values’ (PMP, to distinguish them from log-rank p values (LRP)) indicate the probability of finding a more extreme hazard ratio by chance in the respective tumor type when stratifying patients using the indicated gene-set.

## CFSE labeling

To discriminate proliferating cells from slow or non-diving cells we labelled cells with CFSE (carboxyfluorescein diacetate succinimidyl ester, Sigma), where progeny of proliferating cells lose their green fluorescing CSFE signal while non-dividing or less dividing cells retain it. Briefly, trypsinized cells and cells washed with 10% FCS in DMEM, were resuspended at a concentration of 1 x 10^5^cells/ml in PBS, CSFE at a final concentration of 5µM was added, and incubated for 10min at RT. Labelling was stopped by addition of FBS at a final concentration of 2%, and washed in PBS/2%FCS. Then cells were incubated in adhesion culture as usual. Complete staining of cells was verified using a fluorescent microscope and flow cytometry by detecting the CFSE label in the FITC channel one day after staining.

## Quantitative flow cytometry analysis

To determine cell identity (CSFE label) and cell number simultaneously, we performed ‘quantitative flow cytometry analysis’ by measuring harvested cells in equal volumes under constant flow rates between different samples. Relative cell number of all samples within the indicated experiment (S4 Fig.) is indicated as total number of measured live cells per 60 seconds.

## Statistical methods for single cell analysis

Cross-match test: When using the expression values of both E and M genes statistical analysis was done with a multivariate, nonparametric, two-samples rank test based on an interpoint distance between observations (Rosenbaum, 2005).The two dimensional single cell observations were divided into pairs to minimize the total distance within pairs. The cross-match test statistics is the number of times a cell from the one sample was paired with a cell from the second sample, rejecting the null hypothesis of identical distributions for small values of the statistics. The p-value based on exact null distribution was computed with R package "crossmatch" (<http://cran.r-project.org/web/packages/crossmatch/index.html>).

Mann-Whitman U test: When using the information of either E or M gene sets, we applied the nonparametric Mann-Whitman U test (<http://elegans.som.vcu.edu/~leon/stats/utest.html>) to test the null-hypothesis that two populations came from the same population. Györffy, B., Lanczky, A., Eklund, A.C., Denkert, C., Budczies, J., Li, Q., and Szallasi, Z. (2010). An online survival analysis tool to rapidly assess the effect of 22,277 genes on breast cancer prognosis using microarray data of 1,809 patients. Breast Cancer Res. Treat. *123*, 725–731.

Rosenbaum, P.R. (2005). An exact distribution-free test comparing two multivariate distributions based on adjacency. J R Stat. Soc *67, Part 4*, pp. 515–530.
