# Supplementary figures and images for: Stemness of the hybrid Epithelial/Mesenchymal State in Breast Cancer and Its Association with Poor Survival
Source: PLoS One. 2015 May 28;10(5):e0126522. doi: 10.1371/journal.pone.0126522 (PMC4447403; doi:10.1371/journal.pone.0126522)

S1 Figure

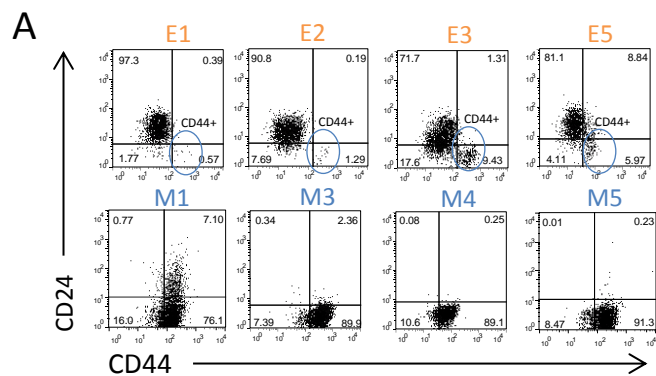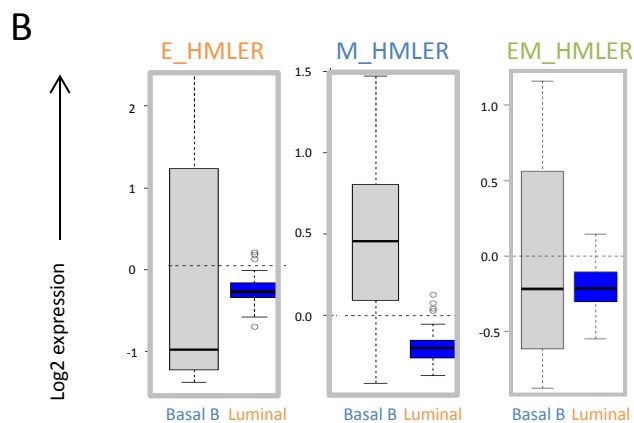

Supplement: S1 Fig — (A) Single cell sorting of parental HMLER cells generated morphologically stable E and M clones. Cells were expanded in adhesion under normal culture conditions. Characteristic CD24/CD44 FACS profiles of clones after about 5–10 passages are shown. Note the small but distinct CD24-/CD44+ (M) populations appearing in all E clones (circled), and the absence of CD24+/CD44- (E) cells in M clones. (B) Comparative expression of E, M, and EM gene sets (S2 Table) relative to basal B or luminal cancer cell lines with expression as assessed by the GOBO online tool (http://co.bmc.lu.se/gobo/) [39]. This classifies E genes as rather luminal-specific and M genes as more basal B-specific genes, and the EM gene set as neutral. The same relative association with E and luminal and M genes and basal cell lines, respectively, was found for the E_HMLER(150) and M_HMLER(150) signatures, as well as for E and M specific gene sets (data not shown) from Taube et al (S2 Table). (PDF) [file pone.0126522.s002.pdf]

S2 Figure

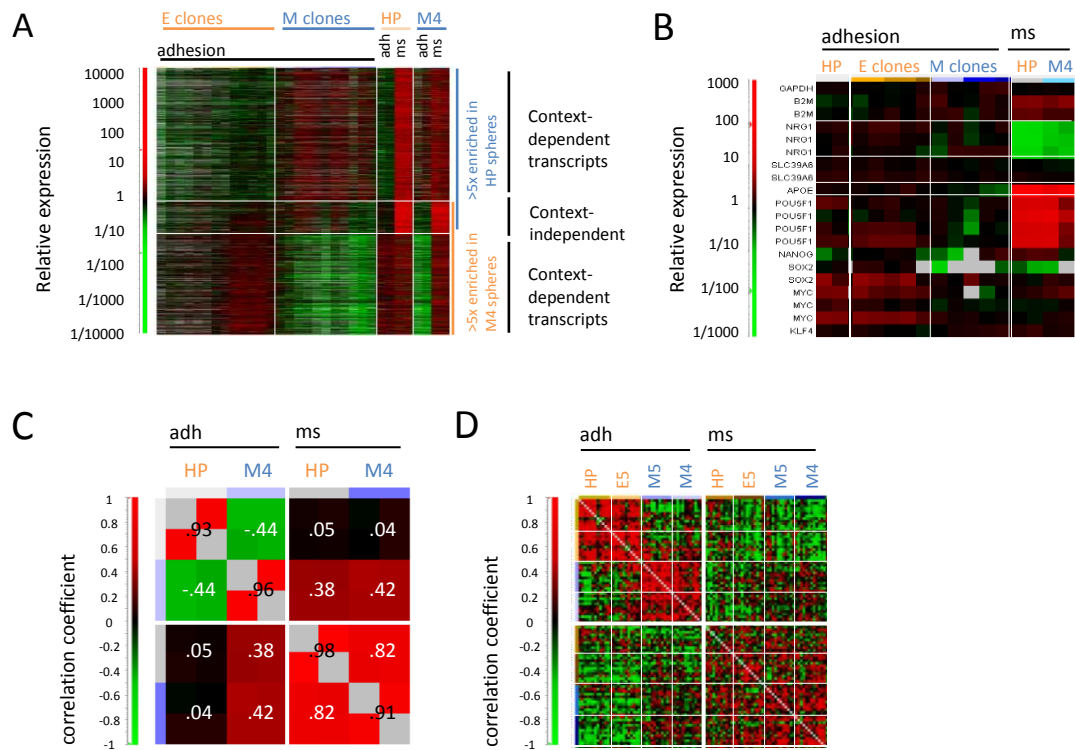

Supplement: S2 Fig — (A,B) Heat maps of gene expression microarray data from adherent cells (adh) and suspended mammospheres (ms) shown after relative normalization towards the intermediate expression in E and M clones. (A) Expression of all transcripts that were enriched >5-fold in suspension grown mammospheres versus the respective adherent cultures. The data show that M genes (expressed higher in adherent M clones) are highly increased in HP mammospheres while E genes (higher expressed in adherent E clones) are increased in M4 mammospheres. Genes that are only enriched in HP mammospheres or only in M4 mammospheres were designated “context-dependent” and the intersection of genes enriched in both cell types as “context-independent transcripts”. (B) Expression of genes expected to be expressed and previously associated with high or low expression in HMEC-derived mammospheres [11]. (C) Pearson correlation for E_HMLER (150) and M_HMLER (150) gene expression in whole population microarrays of HP and M4 cells in adhesion and suspension. (D) Pearson correlation of single cell transcriptomes (Biomark, Fluidigm) measured for 12 cells per group (groups as in C, every column and line per individual cell) and 46 different genes (including, housekeeping genes, E and M-specific genes, pluripotency factor encoding genes, suspension-specific genes) as indicated in S4 Table. Data are from same experiment as shown in Fig 3D. Note the similarity of gene expression correlation in whole population arrays (C) and single cell qPCR analysis: in adhesion we observe a positive correlation between E cells, or between M cells, and anti-correlation between E and M cells, while mammosphere derived cells individual E and M cells cannot be discriminated anymore by correlation. (PDF) [file pone.0126522.s003.pdf]

# S3 Figure

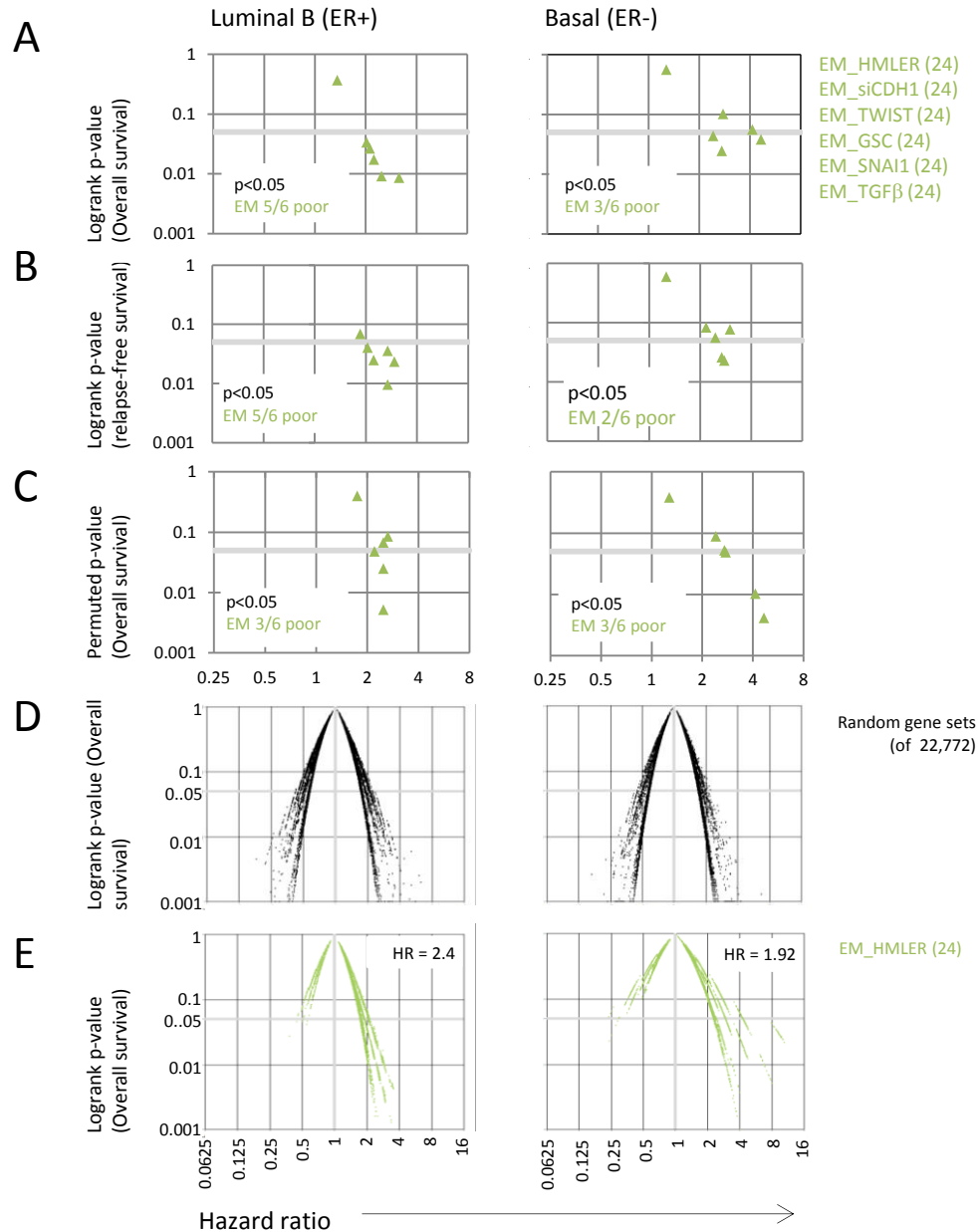

Supplement: S3 Fig — Kaplan-Meier analysis for 6 different EM gene sets (24 genes) from HMLER and HMLE cell lines (S2 Table) in luminal B (epithelial) and basal (mesenchymal) breast cancer patients (Kaplan-Meier-Plotter data set 2010, 72 basal patients, 208 luminal B patients). Numbers of signatures with significant HRs indicating poor prognosis are indicated. Every triangle represents a different gene set. (A) Hazard ratio (HR) for overall survival is plotted against corresponding logrank p-values. HR of >1 indicates poor outcome, HR <1 indicates good outcome. (B) Hazard ratio for relapse-free survival is plotted against corresponding logrank p-values. (C) Hazard ratios for overall survival is plotted against corresponding permuted p-values. Permuted p-values were determined from a permutation test of 106 samples for the respective signature by calculating the probability of achieving a more extreme result with a random gene set of the same size for overall survival of patients. (D) Kaplan–Meier analyses for overall patient survival with gene sets derived by bootstrapping from all 22,772 analyzed probes. 10,000 different gene sets consisting of 10 randomly picked genes were analyzed (every dot represents a different gene set). (E) Kaplan–Meier analyses for overall patient survival with gene sets derived by bootstrapping from the most extreme 24 EM_HMLER genes. 10,000 different gene sets consisting of 10 randomly picked genes were analyzed. (PDF) [file pone.0126522.s004.pdf]

# S4 Figure

A

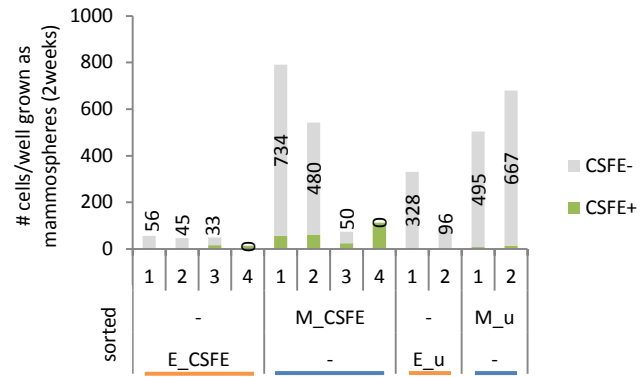

B

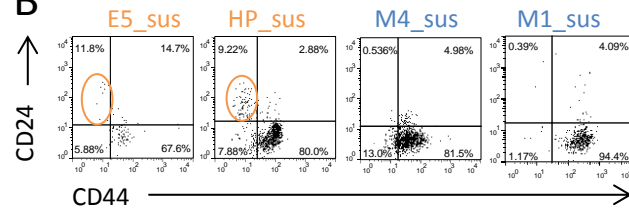

Supplement: S4 Fig — (A) 500 CSFE-positive (CSFE+) cells expressing CD24+ /CD44- (E_CSFE) cells or CD24-/CD44+ M cells (M_CSFE) or 500 CSFE-negative (CSFE-, unlabeled) E and M cells (E_u, M_u) were sorted from HP cells as the indicated biological replicates and cultured in suspension mammosphere conditions for 2 weeks, harvested, and analyzed for their retained CSFE label by quantitative flow cytometry analyses. Bar graphs of relative numbers of CSFE- (proliferating, number indicates CSFE- cell numbers) and CSFE+ (label retaining non-proliferating) cells from dissociated mammospheres per well are shown. Note that the majority of replicates from both E and M cells lost their CSFE label, indicative of proliferation and survival, even though CSFE labeling also appeared toxic to E cells resulting in reduced cell numbers relative to unstained cells from the same source (compare E_CSFE and E_u). (B) Representative CD24/CD44 flow cytometry profiles of dissociated mammospheres (grown 2–3 weeks) from HMLER clones. Note that only cell lines that originally contained E cells (E5 and HP cells) still contained distinct CD24+/CD44- populations but M1 and M4 cells had only a spotty appearance of less than 1% CD24+/CD44- cells, supporting the plasticity of E cells and non-plasticity of M cells. (PDF) [file pone.0126522.s005.pdf]

A

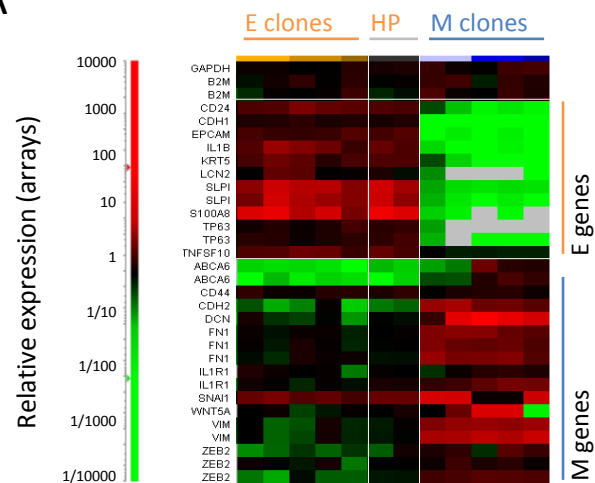

B

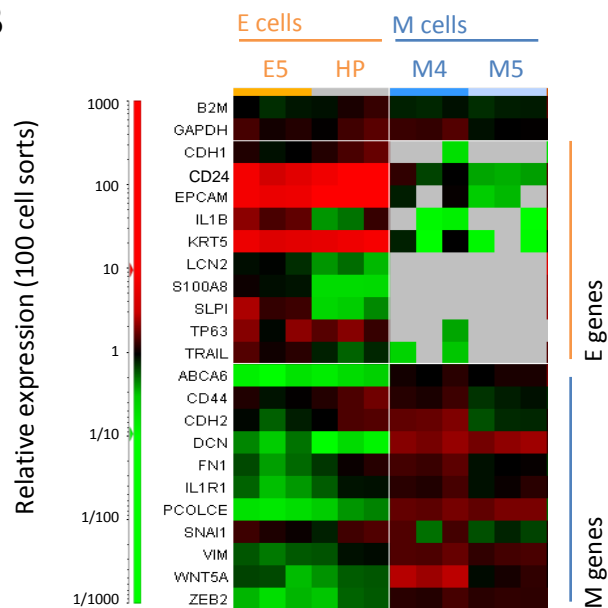

C

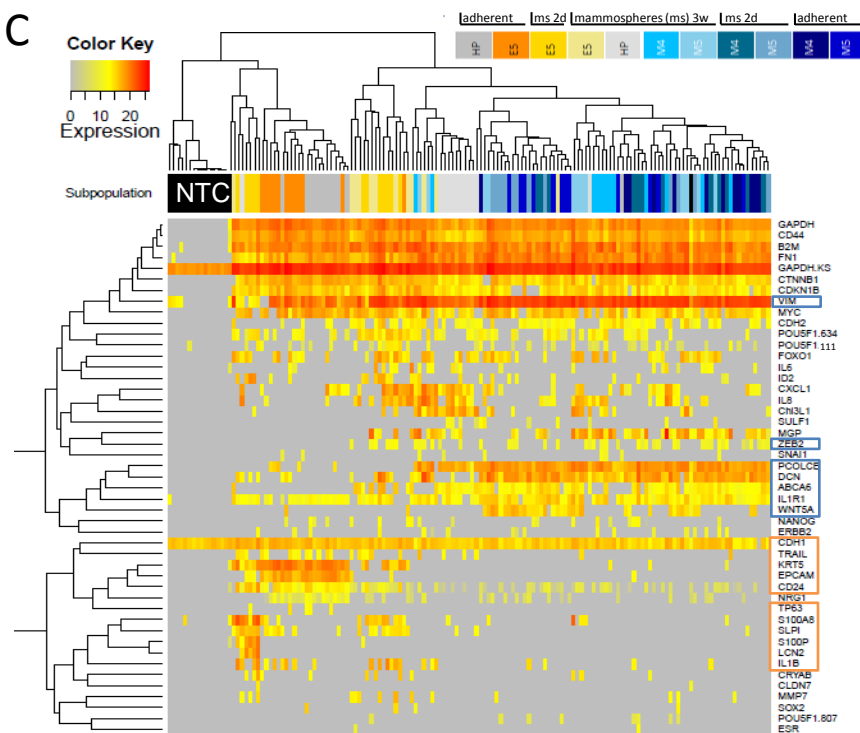

D

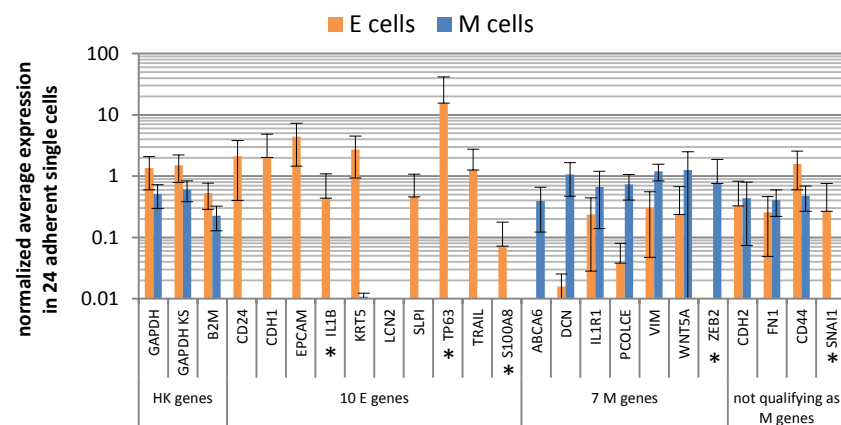

Supplement: S5 Fig — (A) Heat map of selected E and M genes in microarrays of adherent HMLER-derived cell lines (HP cells, 3 different E clones, 3 different M clones with different passage numbers). The same microarray data as used for Fig 1A are shown as relative expression. (B) Heat map of relative gene expression of selected E and M genes assessed by qPCR analysis of 100 AnnexinV-/PI- (live) sorted cells of the indicated sources grown in adhesion (biological triplicates). Using these data we picked E and M genes for single cell qPCR analysis shown in Fig 3D–3F. Note that the usually with the M phenotype associated genes SNAI1, FN, CDH2, and CD44 are not consistently differentially expressed between adherent morphological E and M cell types in arrays and/or qPCR analysis and thus were not used as M-specific genes. (C) Heat map of gene expression in single cells (before subtraction of background) from cell populations grown under adherent or mammosphere suspension conditions as determined by single cell qPCR analysis (same experiment as shown in Fig 3). No-template-controls (empty wells) are designated NTC. Not detected data points are shown in gray. Biclustering based on correlation analysis (using the heatmap.2 R function) of raw expression values including background of adherent and suspended E and M cells for the indicated genes clustered E and M genes into two separate large clusters (the ones used in qPCR analysis are circled in orange and blue), confirming their association with the opposite E and M cell-states. (D) Normalized average expression of individual genes in 24 adherent E cells (HP and E5 clone) versus in 24 adherent M cells (M4 and M5 clones). ‘Not qualifying M genes’ did neither show a biological significant effect-size in arrays, 100 cell- or single cell qPCR analyses. Asterisks mark genes statistically not significant to discriminate between E versus M state. Remarkably, possibly due to the spotty expression of both E and M genes at the single cell level (gray: not [file pone.0126522.s006.pdf]

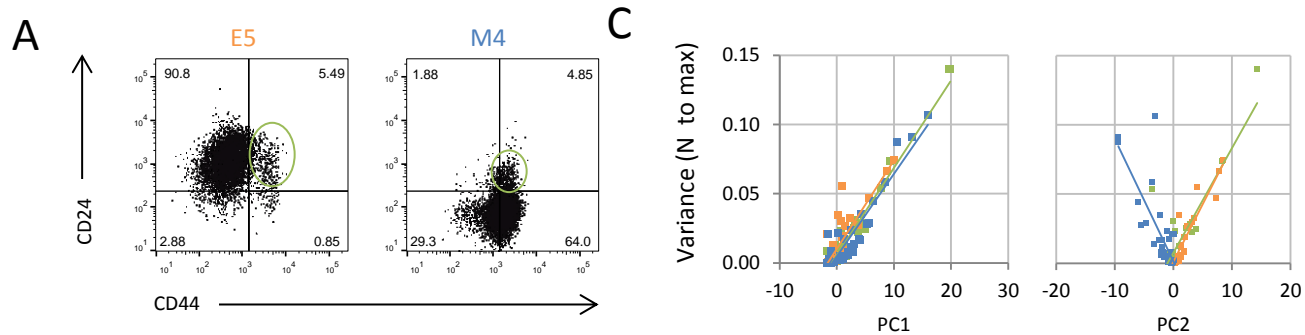

S6 Figure

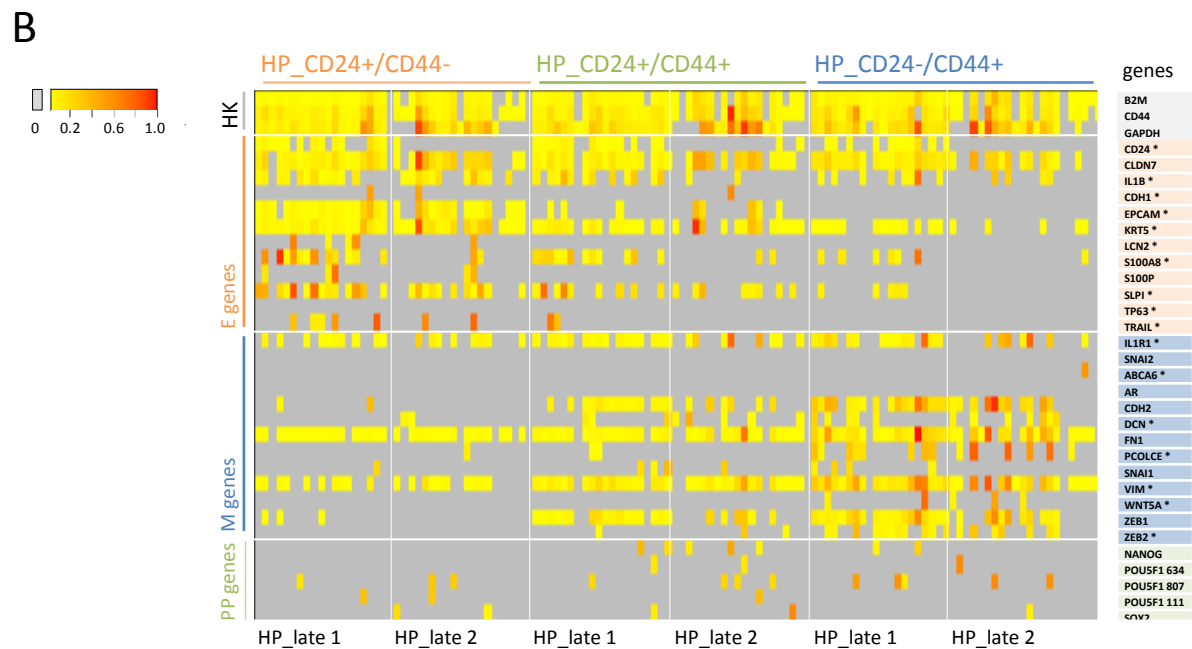

Supplement: S6 Fig — (A) Flow cytometry profile for cell surface CD24 and CD44 expression in HMLER clones E5 and M4 grown in adhesion. Quadrant gate coordinates were determined using simultaneously measured HP cells. (B) Single cell qPCR analysis for two independently passaged HP_late subpopulations (HP_late 1 and HP_late 2). Cells were stained for CD24 and CD44 and 20 cells per indicated cell line and gate were sorted. (C) PC1 and PC2 values of the individual cells (from Fig 5B and 5C) plotted against the variance of expression values per cell (expression value normalized to the maximum expression per gene in whole experiment). Cell subpopulations are labelled according to their CD24/CD44 marker expression during sorting (B). PC1 can be regarded as discriminating between the variance relative to the maximum (diversity), while PC2 was discriminating between E and M cell-types. (PDF) [file pone.0126522.s007.pdf]
