# Supplementary material for: Stemness of the hybrid Epithelial/Mesenchymal State in Breast Cancer and Its Association with Poor Survival
Source: PLoS One. 2015 May 28;10(5):e0126522. doi: 10.1371/journal.pone.0126522 (PMC4447403; doi:10.1371/journal.pone.0126522)
Supplement: S3 Table — GSEA using the indicated E and M gene sets was performed using standard GSEA analysis. Inverse regulation of E genes versus M genes from adhesion to suspension mammosphere cultures was observed, independent whether gene sets were derived from HMLE or HMLER cell lines. (PDF) [file pone.0126522.s010.pdf]

Table S3 - Gene Set enrichment analysis (related to Figure 1E)

		SIZE	ES	NES	NOM p-val	FDR q-val	FWER p-val	RANK AT MAX	LEADING EDGE
HP mammo-spheres versus adhesion	E_HMLER (150)	142	-0.3682842	-5.131866	< 0.0001	< 0.0001	< 0.01	1554	tags=47%, list=11%, signal=52%
	M_HMLER (150)	133	0.6444159	8.585951	< 0.0001	< 0.0001	< 0.01	2296	tags=80%, list=16%, signal=94%
	E_HMLE (128)	141	-0.2024301	-2.8126867	< 0.0001	< 0.0001	< 0.01	1310	tags=29%, list=9%, signal=32%
	M_HMLE (59)	77	0.4747764	4.7592473	< 0.0001	< 0.0001	< 0.01	2001	tags=61%, list=14%, signal=70%
M4 mammo-spheres versus adhesion	E_HMLER (150)	121	0.8161899	10.590222	< 0.0001	< 0.0001	< 0.01	1277	tags=90%, list=9%, signal=98%
	M_HMLER (150)	135	-0.4788928	-6.334316	< 0.0001	< 0.0001	< 0.01	1237	tags=56%, list=9%, signal=61%
	E_HMLE (128)	122	0.6277533	7.952708	< 0.0001	< 0.0001	< 0.01	2978	tags=84%, list=21%, signal=105%
	M_HMLE (59)	77	-0.440355	-4.5861273	< 0.0001	< 0.0001	< 0.01	2404	tags=61%, list=17%, signal=73%
