# Supplementary material for: Stemness of the hybrid Epithelial/Mesenchymal State in Breast Cancer and Its Association with Poor Survival
Source: PLoS One. 2015 May 28;10(5):e0126522. doi: 10.1371/journal.pone.0126522 (PMC4447403; doi:10.1371/journal.pone.0126522)
Supplement: S5 Table — Normalized expression of the average expression of 10 E genes (CDH1, CD24, EPCAM, IL1B, KRT5, LCN2, TP63, TRAIL, SLPI, S100A8) and 7 M genes (ABCA6, DCN, IL1R1, PCOLCE, WNT5A, VIM, ZEB2) per cell was determined as plotted in the EM state space shown in Fig 3E and 3F for the indicated different cell populations all grown in different wells under either adhesion or suspension mammosphere conditions. To examine whether the difference as displayed in the EM state space was statistically significant between the different populations we used the cross-match test (for E and M gene expression) and Mann-Whitney U test for E gene expression or M gene expression. For the single cell data as displayed in Fig 5B, expression data from three different CD24/CD44 cell populations of single cells from adherent HP_late1 and HP_late 2 (plotted in the EM state space shown in Fig 5B) were tested for their differences using the cross-match test and Mann-Whitney U test. (PDF) [file pone.0126522.s012.pdf]

S5 Table: p-values for single cell analyses (related to Figures 3 and 5)

Fig. 3EF

				cross-match	Mann-Whitney U test	
				10 E genes 7 M genes	10 E genes	7 M genes
	24 E_adh	*versus*	24 M_adh	$2.4 * 10^{-5}$	$2 * 10^{-6}$	$2 * 10^{-6}$
	24 E_adh	*versus*	24 E_sus 23d	0.014	$4 * 10^{-6}$	$1 * 10^{-5}$
	24 M_adh	*versus*	24 M_sus 23d	0.014	0.019	0.0056
	24 E_sus 23d	*versus*	24 M_sus 23d	0.30	0.49	0.001
E cells	12 E5_adh	*versus*	12 HP_adh	0.023	0.59	0.84
M cells	12 M4_adh	*versus*	12 M5_adh	0.95	0.76	0.51

Fig. 5B

				cross-match	Mann-Whitney U test	
	HP_late 1/2		HP_late 1/2	10 E genes 7 M genes	10 E genes	7 M genes
	40 CD24+/CD44-	*versus*	40 CD24-/CD44+	$5.5 * 10^{-6}$	$1 * 10^{-5}$	$1 * 10^{-6}$
	40 CD24+/CD44-	*versus*	40 CD24+/CD44+	0.0014	0.042	$7 * 10^{-6}$
	40 CD24-/CD44+	*versus*	40 CD24+/CD44+	0.34	0.0039	0.0024
	HP_late 1		HP_late 2			
E cells	20 CD24+/CD44-	*versus*	20 CD24+/CD44-	0.63	0.023	0.29
EM cells	20 CD24+/CD44+	*versus*	20 CD24+/CD44+	0.89	0.14	0.32
M cells	20 CD24-/CD44+	*versus*	20 CD24-/CD44+	0.29	0.06	0.23
